# Whole genome sequencing and in vitro splice assays reveal genetic causes for inherited retinal diseases

**DOI:** 10.1038/s41525-021-00261-1

**Published:** 2021-11-18

**Authors:** Zeinab Fadaie, Laura Whelan, Tamar Ben-Yosef, Adrian Dockery, Zelia Corradi, Christian Gilissen, Lonneke Haer-Wigman, Jordi Corominas, Galuh D. N. Astuti, Laura de Rooij, L. Ingeborgh van den Born, Caroline C. W. Klaver, Carel B. Hoyng, Niamh Wynne, Emma S. Duignan, Paul F. Kenna, Frans P. M. Cremers, G. Jane Farrar, Susanne Roosing

**Affiliations:** 1 Department of Human Genetics, Radboud University Medical Center, Nijmegen, The Netherlands; 2 Donders Institute for Brain, Cognition and Behavior, Radboud University Medical Center, Nijmegen, The Netherlands; 3 The School of Genetics and Microbiology, Smurfit Institute of Genetics, Trinity College Dublin, Dublin, Ireland; 4 Rappaport Faculty of Medicine, Technion-Israel Institute of Technology, Haifa, Israel; 5 Radboud Institute of Molecular Life Sciences, Radboud University Medical Center, Nijmegen, The Netherlands; 6 Division of Human Genetics, Center for Biomedical Research (CEBIOR), Faculty of Medicine, Diponegoro University, Semarang, Indonesia; 7 The Rotterdam Eye Hospital, Rotterdam, The Netherlands; 8 Department of Ophthalmology, Erasmus Medical Center, Rotterdam, The Netherlands; 9 Department of Epidemiology, Erasmus Medical Center, Rotterdam, The Netherlands; 10 Department of Ophthalmology, Radboud University Medical Center, Nijmegen, The Netherlands; 11 Department of Ophthalmology, Royal Victoria Eye and Ear Hospital, Dublin, Ireland

**Keywords:** Next-generation sequencing, RNA splicing, Retinal diseases

## Abstract

Inherited retinal diseases (IRDs) are a major cause of visual impairment. These clinically heterogeneous disorders are caused by pathogenic variants in more than 270 genes. As 30–40% of cases remain genetically unexplained following conventional genetic testing, we aimed to obtain a genetic diagnosis in an IRD cohort in which the genetic cause was not found using whole-exome sequencing or targeted capture sequencing. We performed whole-genome sequencing (WGS) to identify causative variants in 100 unresolved cases. After initial prioritization, we performed an in-depth interrogation of all noncoding and structural variants in genes when one candidate variant was detected. In addition, functional analysis of putative splice-altering variants was performed using in vitro splice assays. We identified the genetic cause of the disease in 24 patients. Causative coding variants were observed in genes such as *ATXN7*, *CEP78*, *EYS*, *FAM161A*, and *HGSNAT*. Gene disrupting structural variants were also detected in *ATXN7*, *PRPF31*, and *RPGRIP1*. In 14 monoallelic cases, we prioritized candidate noncanonical splice sites or deep-intronic variants that were predicted to disrupt the splicing process based on in silico analyses. Of these, seven cases were resolved as they carried pathogenic splice defects. WGS is a powerful tool to identify causative variants residing outside coding regions or heterozygous structural variants. This approach was most efficient in cases with a distinct clinical diagnosis. In addition, in vitro splice assays provide important evidence of the pathogenicity of rare variants.

## Introduction

Inherited retinal diseases (IRDs) are a group of conditions showing dysfunction and/or degeneration of the neural retina or retinal pigment epithelium, resulting in visual impairment. They affect more than two million people worldwide and display a very high degree of clinical and genetic heterogeneity^1,2^. Therefore, defining the precise underlying genetic cause of the disease has a profound impact on the potential to diagnose, counsel, and provide accurate family risk assessment. Furthermore, genetic diagnoses can enable the development of new therapeutic approaches and access to these treatments^2–4^.

High-throughput DNA sequencing technologies are widely used to investigate IRD pathogenesis as they display a high degree of heterogeneity, with more than 270 genes and loci associated with IRDs, encompassing all Mendelian inheritance patterns and digenic inheritance (https://sph.uth.edu/retnet/). Next-generation sequencing (NGS) facilitates the analysis of genetic variation in multiple regions of the genome in a single experiment^5^. For many years, the application of NGS in IRD molecular diagnostics had been limited to target-capture sequencing (TCS) and whole-exome sequencing (WES). TCS has been shown to be an effective preliminary diagnostic technique, boasting cost effectiveness, rapid data generation, and lower volumes of data interpretation as major advantages^6–8^. Sequence analysis of only previously IRD-associated genes is one of the major disadvantages of this technique^9,10^. On the contrary, WES is not limited to IRD-associated genes, as this approach also enables the analysis of genes not yet associated with IRD. It is estimated that 85% of pathogenic variants are located within protein-coding regions, the primary focus of WES^11,12^. However, this may vary depending on the particular disease entity. It is evident that sequencing noncoding regions of the genome is required to also identify causal deep-intronic variants and to improve the identification of structural variants (SVs) and their breakpoints^13–16^.

An accurate splicing process requires highly conserved sequences in the canonical splice donor site (SDS) and splice acceptor site (SAS). All SASs contain AG at the canonical −1 and −2 positions and 98.7% of SDSs contain GT in the canonical +1 and +2 positions. In addition, the first and last three nucleotides of an exon as well as the −3 to −14 nucleotides from the SAS and +3 to +6 of the SDS are conserved and ensure a precise splicing process^17^. However, variants in intronic regions of the genome can introduce or strengthen a cryptic splice site, which resembles a canonical splice site, and in the presence of a nearby SAS or SDS, may lead to pseudoexon insertion and potentially disrupt the reading frame causing protein truncation^17,18^. In both TCS and WES approaches, the analysis is restricted to genetic variations in protein-coding and flanking splice site regions of the genome. Furthermore, these techniques are limited in their ability to detect SVs such as deletions, duplications, or inversions when breakpoints lie in intronic or intergenic regions^19^.

These limitations are less evident in WGS, which enables the identification of most types of variation across the entire genome^2,20^. TCS and WES are the most commonly employed techniques for the genetic diagnosis of IRDs. However, because of decreasing costs and increasing data interpretability, WGS is becoming an attractive alternative albeit predominantly for research purposes, as diagnostic laboratories are able to interpret variants only in previously associated IRD genes with confidence using the American College of Medical Genetics and Genomics (ACMG) guidelines^19^. In addition, IRD cases are ideal candidates for WGS analysis as they are largely presumed to be monogenic diseases with a high degree of genetic heterogeneity.

In this study, we aimed to investigate the sensitivity and accuracy of WGS in a cohort of 100 individuals with different IRDs. All individuals were previously tested by TCS or WES, but a genetic cause of disease remained undetermined. The study provides insights into the increased resolution of IRD cases that can be achieved by employing WGS.

## Results

### Patient characterization

We performed WGS on 100 IRD cases with a suspected autosomal recessive inheritance recruited in Ireland, Israel, and The Netherlands. Among these, TCS was previously performed on 38 Irish and eight Israeli cases, which did not result in a complete genetic diagnosis. The remaining 54 cases (43 Dutch and 11 Israeli) were previously examined using WES and also remained genetically unresolved. In 56/100 cases, a first plausible pathogenic variant was selected from prior WES or TCS data based on a minor allele frequency of <1% in the ExAC database^21^ and 5% in dbSNP as well as <1% in an in-house database of 15,576 mostly Caucasian individuals and <2% in an in-house database of 454 mostly Asian individuals^22^. In the prior prioritization of monoallelic cases, all coding and splice site variants (nucleotides −17 to −1 and +1 to +6) were manually classified according to a five-class system based on the ACMG-Association for Molecular Pathology (AMP) classification system^23^, followed by evaluating the prioritized variants using practice guidelines by Wallis et al.^24^. Therefore, these were included as monoallelic cases in this study (Supplementary Table 1).

**Fig. 1:**
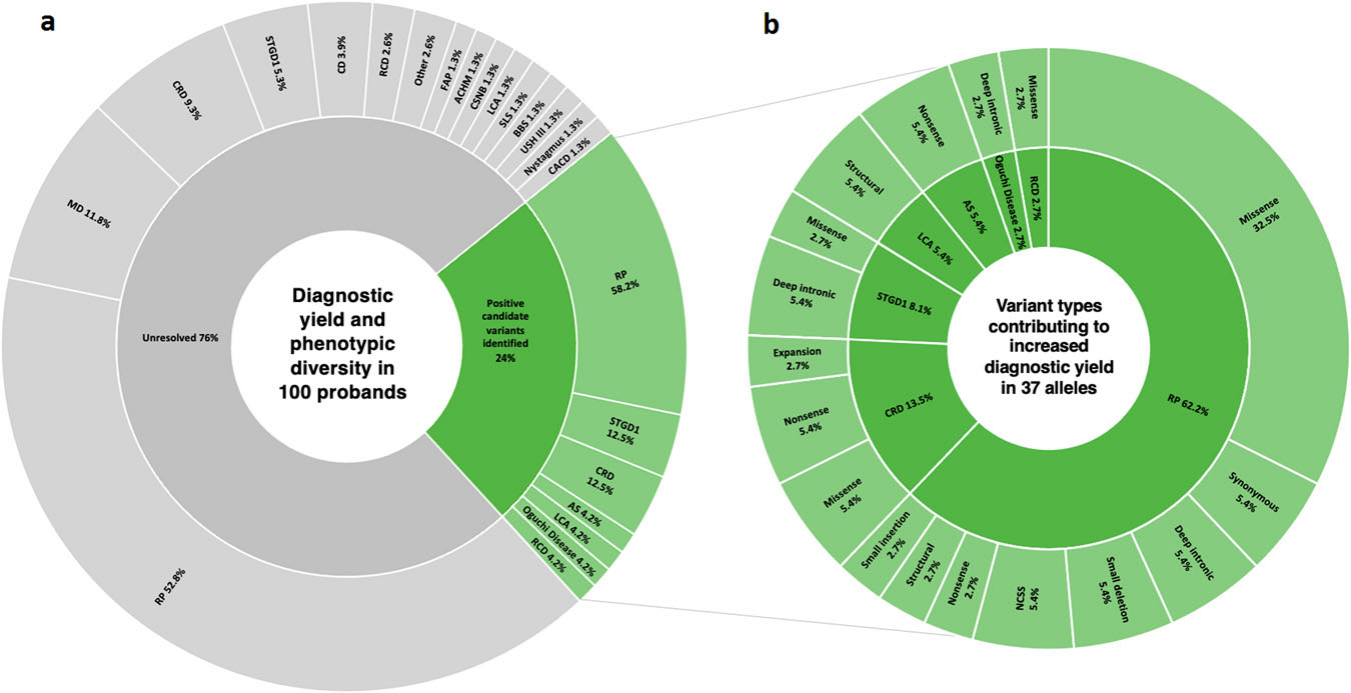
Sunburst diagrams illustrating diagnostic yield, phenotypic diversity and variants contributing to increased diagnostic yield in this study. **a** Diagnostic yield and phenotypic diversity. The inner rings represent solved (green) and unresolved (gray) cases following whole-genome sequencing. The outer ring encompasses the phenotypic presentation of patients in these groups. **b** Variant types contributing to increased diagnostic yield. The types of variants contributing to increased diagnostic yield are illustrated on the outer ring, with their corresponding clinical phenotypes on the inner ring. ACHM achromatopsia, AS Alström syndrome, BBS Bardet–Biedl syndrome, CACD central areolar choroidal dystrophy, CD cone dystrophy, CRD cone–rod dystrophy, CSNB congenital stationary night blindness, FAP fundus albipunctatus, LCA Leber congenital amaurosis, MD macular dystrophy, NCSS noncanonical splice site, RCD rod–cone dystrophy, RP retinitis pigmentosa, SLS Senior–Loken syndrome, STGD1 Stargardt disease type 1, USH III Usher syndrome type III. Small insertions include insertions <4-bp in length. Small deletions include deletions <4-bp in length.

A broad range of IRDs were included in this cohort (Table 1 and Fig. 1). Retinitis pigmentosa (RP, OMIM: 268000) is the most frequently listed phenotype (53%), followed by cone–rod dystrophy (9%, OMIM: 601777), macular dystrophy (8%, OMIM: 616152), and Stargardt disease (STGD1, 6%, OMIM: 248200). Rare forms of IRD such as Leber congenital amaurosis (LCA, OMIM: 204000), Bardet–Biedl syndrome (OMIM: 209900), and Senior–Løken syndrome (OMIM: 266900) were also included.

**Table 1:** Phenotypic diversity of patients and the number of cases referred for TCS, WES, and WGS.

Clinical diagnosis	No. of cases previously tested by WES	No. of cases previously tested by TCS	No. of cases tested by WGS
Retinitis pigmentosa	21	32	53
Cone dystrophy	2	1	3
Rod–cone dystrophy	3	0	3
Cone–rod dystrophy	7	2	9
Alström syndrome	0	1	1
Bardet–Biedl syndrome	1	0	1
Leber congenital amaurosis	2	0	2
Macular dystrophy	8	1	9
Oguchi disease	1	0	1
Central areolar choroidal dystrophy	1	0	1
Fundus albipunctatus	1	0	1
Usher syndrome type III	1	0	1
Senior–Løken syndrome	1	0	1
Nystagmus	1	0	1
Stargardt disease	0	7	7
Clumped pigmentary retinal dystrophy	1	0	1
Cone–rod dystrophy with progressive neurodegeneration	1	0	1
Retinitis pigmentosa with hypogonadism	1	0	1
Bietti crystalline corneoretina dystrofie	1	0	1
Congenital stationary night blindness	0	1	1
Achromatopsia	0	1	1
Total number	54	46	100

*WES* whole-exome sequencing, *TCS* target-capture sequencing, *WGS* whole-genome sequencing.

### Identification of coding and noncoding candidate variants after WGS data analysis

Following comprehensive WGS analysis and in vitro functional assays, we identified a genetic cause of disease in 24 probands (24%). Pathogenic variants were confirmed by Sanger sequencing and segregation analysis when family members were available (9/24) (Table 2 and Supplementary Table 2). Among these, 15 probands (55.5%) carried previously undetected pathogenic variants in coding regions and three individuals (11.1%) carried SVs in *RPGRIP1* and *PRPF31*, or a triplet tandem repeat expansion in *ATXN7*. In proband Pt-4, presenting with cone–rod dystrophy and progressive neurodegeneration (OMIM: 164500), we identified a pathogenic CAG repeat expansion in exon 3 of *ATXN7* (NG_008227.1, p.(Gln30[70])) (Table 2 and Supplementary Fig. 1A). While the wild-type allele carries 3–19 CAG repeats in *ATXN7*, the pathogenic allele can be between 37 and 460 repeats^25^.

**Table 2:** Overview of pathogenic variants in genetically solved cases.

ID	Ethnicity	Phenotype	Gene	DNA allele 1	Protein allele 1	DNA allele 2	Protein allele 2	Previous genetic testing method	Segregation analysis, # of family tested members
Pt-1	Irish	STGD1	*ABCA4*	c.6079C>T^b^	p.(Leu2027Phe)	c.5196+1137G>A	p.[=,Met1733Glufs*78]	TCS	No
Pt-2	Irish	STGD1	*ABCA4*	c.6743T>C^b^	p.(Phe2248Ser)	c.4539+2028C>T	p.[=,Arg1514Leufs*36]	TCS	No
Pt-3	Irish	STGD1	*ABCA4*	c.5603A>T^b^	p.(Asn1868Ile)	c.286A>C	p.(Asn96His)	TCS	No
Pt-4	Dutch	CRD^a^	*ATXN7*	c.88_117CAG[70]	p.(Gln30[70])	–	–	WES	Yes, 2
Pt-5	Irish	RP	*C21ORF2*	c.218G>C	p.(Arg73Pro)	c.76T>C	p.(Trp26Arg)	TCS	No
Pt-6	Irish	RP	*C21ORF2*	c.218G>C	p.(Arg73Pro)	c.218G>C	p.(Arg73Pro)	TCS	No
Pt-7	Irish	RP	*CDHR1*	c.783G>A	p.Asp214_Pro261del	c.783G>A	p.Asp214_Pro261del	TCS	No
Pt-8	Israeli	AS	*CEP78*	c.1033G>T	p.(Gly345*)	c.1033G>T	p.(Gly345*)	TCS	No
Pt-9	Dutch	RP	*EYS*	c.[3906C>A;9468T>A]^b^	p.[Tyr3156*;His1302Gln]	c.5644+70912A>G	p.Asp1882Glyfs*18	WES	Yes, 1
Pt-10	Israeli	RP	*EYS*	c.403_423delinsCTTTT^b^	p.(Thr135Argfs*26)	c.539C>A	p.(Ser180*)	TCS	Yes, 1
Pt-11	Israeli	CRD	*EYS*	c.9468T>A	p.(Tyr3156*)	c.9468T>A	p.(Tyr3156*)	WES	No
Pt-12^c^	Dutch	RCD	*EYS*	c.1660T>A^b^	p.(Cys554Ser)	c.5044G>T	p.(Asp1682Tyr)	WES	No
Pt-13	Israeli	RP	*FAM161A*	c.1753_1756del	p.(Lys585Alafs*6)	c.1753_1756del	p.(Lys585Alafs*6)	TCS	No
Pt-14	Dutch	Oguchi disease	*GRM6*	c.1732C>T^b^	p.(Arg578Cys)	c.1355-587dup	p.[Asn451_Ala452ins25,Gly385Alafs*42,=]	WES	No
Pt-15	Dutch	RP	*HGSNAT*	c.1843G>A	p.Ala615Thr	c.1843G>A	p.Ala615Thr	WES	No
Pt-16	Dutch	RP	*HGSNAT*	c.1030C>T	p.(Arg344Cys)	c.1843G>A	p.Ala615Thr	WES	No
Pt-17	Dutch	RP	*HGSNAT*	c.1843G>A^b^	p.Ala615Thr	c.493+5G>A	p.Arg124Serfs*25	WES	No
Pt-18	Dutch	RP	*HGSNAT*	c.1843G>A	p.Ala615Thr	c.1843G>A	p.Ala615Thr	WES	No
Pt-19	Irish	RP	*HGSNAT*	c.1622C>T	p.(Ser541Leu)	c.1843G>A	p.Ala615Thr	TCS	Yes, 3
Pt-20	Irish	RP	*PCARE*	c.3604C>T^b^	p.(Arg1202*)	c.3099_3100insCAGG	p.(Val1034Glnfs*74)	TCS	Yes, 5
Pt-21	Israeli	CRD	*RGS9BP*	c.583T>G	p.(Ser195Ala)	c.583T>G	p.(Ser195Ala)	WES	No
Pt-22	Dutch	LCA	*RPGRIP1*	c.3238+1118_3339+323del	p.(Asp1080Glyfs*6)	c.3238+1118_3339+323del	p.Asp1080Glyfs*6	WES	Yes, 2
Pt-23	Irish	RP	*PRPF31*	c.-396_*287(0) (g.54106454-54133135del)	p.(0)	–	–	TCS	Yes, 4
Pt-2	Israeli	RP	*USH2A*	c.4758+3A>G	p.Gln1586_Gly1587ins*5	c.784+14389G>T	p.Gly262Aspfs*26	TCS	No

*ABCA4*: NG_009073.1; *ATXN7*: NG_008227.1; *C21ORF2*: NG_032952.1; *CDHR1*: NG_028034.1; *CEP78*: NG_053171.1; *EYS*: NG_023443.2; *FAM161A*: NG_028125.1; *GRM6*: NG_008105.1; *HGSNAT*: NG_009552.1; *PCARE*: NG_021427.1; *RGS9BP*: NG_016751.1; *RPGRIP1*: NG_008933.1; *PRPF31*: NG_009759.1; *USH2A*: NG_009497.2.

*AS* Alström syndrome, *CD* cone dystrophy, *CRD* cone–rod dystrophy, *LCA* Leber congenital amaurosis, *RCD* rod–cone dystrophy, *RP* retinitis pigmentosa, *TCS* target-capture sequencing, *WES* whole-exome sequencing.

^a^With neurodegeneration.

^b^A first pathogenic allele was identified in previous genetic testing.

^c^This patient is assigned as likely solved.

The second proband (Pt-22) illustrates the strength of WGS for SV detection compared to WES. The patient was diagnosed with LCA (MIM: 605446), with one pathogenic allele in *CEP290* (p.(Arg360Gln)), *PDE6A* (p.(Val685Met)), and *RPE65* (p.(Val226Ile)) (Supplementary Table 1). However, WGS data revealed—and segregation analysis confirmed—a homozygous *RPGRIP1* (NG_008933.1) exon 20 deletion (p.(Asp1080Glyfs*6)), missed previously due to incapability of WES analysis tools to detect copy number variants (CNVs) using the SureSelect XT Human All Exon V4 (SOLiD 5500xl sequencer in 2012). While we cannot rule out that a more recent WES strategy would have detected this single exon deletion, this approach would not have revealed the breakpoints. A 12-bp microhomology region was identified in the breakpoint boundaries (Table 2 and Supplementary Fig. 1D). Completing the variant analysis, the noncoding regions of *CEP290*, *RPE65*, and *PDE6A* did not contain additional potential pathogenic variants. The third proband (Pt-23) had a 26.68-kb heterozygous deletion on chromosome 19, encompassing *PRPF31*, *TFPT*, and the promoter region of *NDUFA3*. This large deletion was not detected previously using TCS assessment (Table 2 and Supplementary Fig. 1B).

Furthermore, we prioritized ten deep-intronic variants and three noncanonical splice site (NCSS) variants in 14 individuals using criteria in Alamut and SpliceAI (Supplementary Tables 3 and 4) outlined in the variant frequency and pathogenicity prediction parameters section of the methods. For the indel variants detected in *ABCA4* (NG_009073.1, in Pt-28, Pt-29, and Pt-30), *CYP4V2* (NG_007965.1, in Pt-52), and *RLBP1* (NG_008116.1, in Pt-65), no SpliceAI scores were provided due to the nature of these variants. Therefore, inclusion of these three variants for in vitro splice assays was based on Alamut prediction scores only. The pathogenicity of noncoding variants was determined in seven cases (46.6%) after in vitro splice assays (see below) (Table 2 and Fig. 1). While employing WGS, significant progress was made in defining the genetic pathogenesis of IRDs in this prescreened IRD cohort; 76% of cases remained genetically unexplained.

### Splice defects due to NCSS variants in *CDHR1*, *HGSNAT*, and *USH2A*

In Pt-7, we identified a homozygous synonymous variant in the last nucleotide of *CDHR1* exon 8, c.783G>A (NG_028034.1). In 2017, Stingl et al. reported that a patient was homozygous for the same variant. A midigene splice assay encompassing exons 7, 8, and 9 demonstrated that this variant causes in-frame skipping of exon 8 (p.Asp214_Pro261del)^26^. Likewise, a similar defect was identified in messenger RNA (mRNA) from the retina of an individual heterozygous for this variant. The authors suggest that this variant is associated with a relatively mild form of IRD, based on clinical examination of a patient homozygous for this variant^26^. Similarly, our patient displays a mild phenotype and late age-at-onset as previously described^26^.

**Fig. 2:**
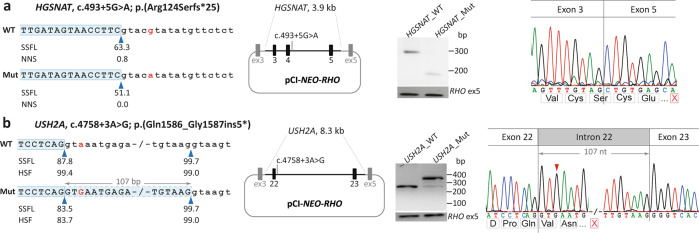
Splice defects due to noncanonical splice site variants. For *HGSNAT* c.493+5G > A (**a**) and *USH2A*, c.4758+3A>G (**b**), the left panels are schematic representations of the gene segments of interest containing the corresponding variants in the wild-type (WT) and mutant (Mut) sequences. The SpliceSiteFinder‐like (SSFL, range 0–100), and Human Splicing Finder (HSF, range 0–100) or NNSPLICE (NNS, range 0–1) scores for the splice sites are indicated below each sequence. The blue triangles indicate the position of splice donor sites in both wild-type and mutant sequences. The red highlighted nucleotides indicate the variants in the mutant sequence. The second panels show the mutant pCI‐*NEO‐RHO* vectors. They contain the variants and flanking exons 3, 4, and 5 in *HGSNAT* and exons 22 and 23 in *USH2A* flanked by *RHO* exons 3 and 5. Wild-type and mutant constructs were used to transfect HEK293T cells. The next panels show the gel images of RT-PCR products in wild-type and mutant midigenes. RT‐PCR analysis of *RHO* exon 5 was performed as a control for transfection efficiencies. In the last panels, the Sanger sequence analysis of the RT‐PCR fragments confirmed the exon 4 skipping in *HGSNAT*, c.493+5G>A (**a**) and 107-nt exon elongation in *USH2A* c.4758+3A>G (**b**). The red “X” in a red box indicates the stop codon in each schematic chromatogram. For each variant, both WT and mutant variants were examined in parallel in the same experiment and processed in parallel.

In Pt-17, a pathogenic missense variant (c.1843G>A) was identified in *HGSNAT* (NG_009552.1) as the first allele. Through WGS analysis, a NCSS variant, c.493+5G>A, was identified in the same gene, resulting in exon 4 skipping (p.Arg124Serfs*25) as evaluated using a midigene splice assay spanning exons 3, 4, and 5 (Fig. 2a). The variant was classified as severe due to the absence of remaining wild-type mRNA when testing the mutant construct.

Individual Pt-24 with autosomal recessive RP carried a heterozygous variant, c.4758+3A>G in *USH2A* (NG_009497.2) together with a deep-intronic variant, c.784+14389G>T, in the same gene. The NCSS variant was found to be pathogenic after performing a midigene splice assay showing exon elongation (p.Gln1586_Gly1587ins5*) (Fig. 2b). Two fragments were observed upon transfection of the mutant midigene in human embryonic kidney 293 T (HEK293T) cells. After quantification, we observed 78.9% of a larger fragment (332 nt) corresponding to 107-bp elongation of exon 22 and 21.1% of the wild-type fragment (225 nt) enabling classification of this variant as severe (Supplementary Table 5).

### Splice defects due to deep-intronic variants in *ABCA4*, *EYS*, *GRM6*, and *USH2A*

**Fig. 3:**
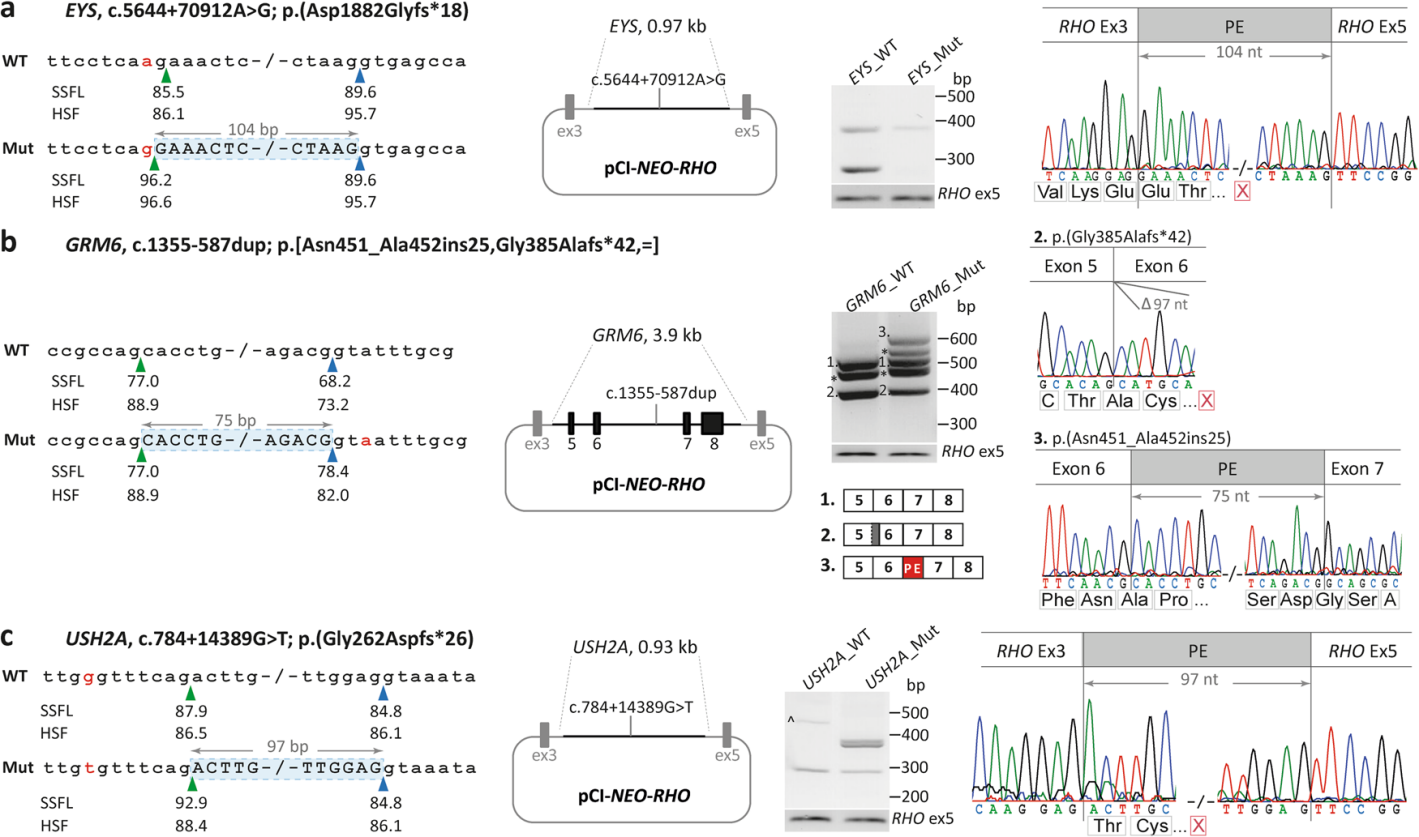
Splice defects due to deep-intronic variants. For each variant, i.e., **a** *EYS*, c.5644+70912A>G, **b** *GRM6*, c.1355-587dup, and **c** *USH2A*, c.784+14389G>T, the left panels are schematic representations of the gene segments of interest containing the corresponding variants in the wild-type (WT) and mutant (Mut) sequences. The SpliceSiteFinder‐like (SSFL, range 0–100), and Human Splicing Finder (HSF, range 0–100) scores for the splice sites are indicated below each sequence. The green and blue triangles indicate the position of splice acceptor sites and splice donor sites in both wild-type and mutant sequences. The red highlighted nucleotides indicate the variants in the mutant sequence. The second panels show the mutant pCI‐*NEO‐RHO* vectors. They contain the variants and flanking exons (except for *EYS*, as c.5644+70912A>G is in a minigene construct) flanked by *RHO* exons 3 and 5. Wild-type and mutant constructs were used to transfect HEK293T cells. The next panels show the gel images of RT-PCR products in wild-type and mutant midi- or minigenes. RT‐PCR analysis of *RHO* exon 5 was performed as a control for transfection efficiencies. For the *GRM6* c.1355-587dup variant, the asterisks in the gel image indicate the heteroduplex fragments. For the *USH2A* c.784+14389G>T variant, the “^” in the wild-type construct indicates an artifact product. In the last panels, the Sanger sequence analysis of the RT‐PCR fragments confirmed the predicted splice defects for each variant. For *GRM6* c.1355-587dup, the sequence of fragment 2 in both midigenes showed the 97-bp deletion of exon 6, which led to the frameshift variant. The third fragment was only observed in the mutant construct and corresponded to the insertion of an in-frame pseudoexon in intron 6. The red “X” in a red box indicates the stop codon in each schematic chromatogram. For each variant, both WT and mutant variants were examined in parallel in the same experiment and processed in parallel.

In the aforementioned Pt-24, after confirming the pathogenicity of the c.4758+3A>G NCSS variant in *USH2A*, an extensive analysis of the intronic regions of this gene was performed and a deep-intronic variant, c.784+14389G>T, was detected in *trans* in *USH2A* that led to a pseudoexon insertion (p.Gly262Aspfs*26) (Fig. 3c). Using the mutant construct for midigene analysis, 77.1% of the mutant fragment (371 nt) corresponding to the presence of a pseudoexon in mature mRNA was observed and 22.9% of the wild-type fragment (274 nt). Therefore, this allele was classified as severe (Supplemental File 1: Table 5). This patient also carried a frameshift variant in *PCARE* (p.(Lys919Thrfs*2)), which was detected previously by TCS. However, WGS analysis did not reveal any plausible second candidate variant in intronic regions of the *PCARE* gene (Supplemental File 1: Table 1).

Two STGD1 cases (Pt-1 and Pt-2) carried c.6079C>T and c.674T>C missense variants, individually, as pathogenic alleles (Supplementary Table 1). Upon WGS analysis, we identified previously published pathogenic deep-intronic *ABCA4* variants in these probands, c.4539+2028C>T and c.5196+1137G>A, respectively^27^. The c.4539+2028C>T variant did not show a splice defect in fibroblast cells, but revealed a retina-specific 345-nt pseudoexon insertion in a proportion of the mRNA (p.[=,Arg1514Leufs*36]) extracted from patient-derived photoreceptor precursor cells^4^. The variant c.5196+1137G>A showed a 73-nt pseudoexon insertion in a small fraction of mRNA in a HEK293T cell–midigene-based splice assay. This effect was stronger in patient-derived photoreceptor precursor cells (p.[=,Met1733Glufs*78])^28^. Based on genotype–phenotype correlations, variants c.4539+2028C>T and c.5196+1137G>A were deemed moderately severe^27,28^.

*EYS* c.5644+70912A>G (NG_023443.2) was identified in Pt-9, who carried c.[3906C>A;9468T>A] (p.[His1302Gln;Tyr3156*], NM_001292009.1) in *EYS* as the first pathogenic allele for which the truncating variant is mostly reported as c.9405T>A (p.(Tyr3135*), NM_001142800.1) (Supplementary Table 1). Due to the large size of intron 26 (150.8 kb), c.5644+70912A>G was tested in a minigene with a small genomic DNA insert (0.97 kb; Fig. 3a). Reverse transcription-polymerase chain reaction (RT-PCR) analysis of the mRNA resulting from the wild-type *EYS* minigene revealed the expected wild-type fragment of 274 nt (76.0% of the total product) and a larger fragment of 378 nt (24.0% of the total product) corresponding to the presence of a 104-nt pseudoexon, which leads to frameshift variant after 18 amino acids (p.Asp1882Glyfs*18). mRNA products from the mutant *EYS* c.5644+70912A>G minigene only contained the larger fragment (378 nt), with no remaining wild-type mRNA, and therefore the allele was classified as severe (Fig. 3a and Supplementary Table 5).

In Pt-14, who was monoallelic for *GRM6*, c.1732C>T (NG_008105.1; Supplementary Table 1), *GRM6* c.1355-587dup was identified, which resulted in the insertion of an in-frame pseudoexon encoding 25 amino acids (p.Asn451_Ala452ins25) (Fig. 3b). Surprisingly, a 97-nt partial deletion of exon 6 (359 nt) in both wild-type and mutant midigene assays was observed, which was calculated to be 54.4% and 37.0% of total RNA molecules, respectively. In the *GRM6* mutant midigene assay, the 567-nt fragment corresponding to the presence of a pseudoexon was quantified as 21.0% of total mRNA molecules. The percentage of full-length wild-type fragments (492 nt) for wild-type and mutant *GRM6* midigene assays were 45.6% and 42.0%, respectively. Therefore, we normalized the percentage of wild-type fragment in the assays using the mutant construct (42.0%) to the wild-type construct (46.0%). *GRM6* c.1355-587dup was classified as a mild allele due to the remaining 91.0% of the wild-type fragment (42/46 × 100) (Supplementary Table 5).

Finally, five noncoding variants in seven cases did not show any splice defects after midi- or midigene in vitro splice assays and we therefore classify these as likely benign (Supplementary Fig. 2A–D): *ABCA4*, c.6148-89G>A in Pt-27; *ABCA4*, c.5460+1315_5460+1317delinsTA in three cases (Pt-28–30); *CYP4V2*, c.214+879_214+882delinsG in Pt-52; *PDE6B* c. 469-776C>G in Pt-61; *RLBP1* c.525+425_525+433delinsATA in Pt-65. These patients were included in this study as monoallelic cases having the first pathogenic allele in *ABCA4*, *CYP4V2*, *PDE6B*, and *RLBP1*, respectively (Supplementary Table 1). However, all seven probands remained genetically unresolved given that a potential pathogenic second allele was not identified.

## Discussion

The sophistication of sequencing methods employed for the detection of disease-causing variants in the human genome has increased rapidly over the past decade, from targeted approaches such as panel sequencing to more comprehensive diagnostic methods such as WES and WGS. TCS and WES have undoubtedly played a valuable role in the genetic diagnosis of IRDs. For genetically undiagnosed cases following TCS and WES, it has become clear that more comprehensive sequencing procedures are required. Importantly, WGS facilitates the identification of noncoding variants, now widely considered a prominent cause of a wide spectrum of IRDs^18,19,29–33^. In order to investigate these possible advantages, we performed WGS for 100 probands with clinical indications of IRD who had received inconclusive results from TCS and WES previously.

This study cohort consisted of 56 monoallelic individuals who carried one candidate pathogenic variant and 44 individuals with no candidate pathogenic variants previously identified in an IRD-associated gene via WES or TCS (Supplementary Table 1). Following extensive WGS data analysis and employment of in vitro splice assays, we identified the genetic cause of disease in 24 cases. These findings demonstrate that WGS is a more comprehensive sequencing method than TCS and WES for IRDs. As previously described in the literature, we have also observed a higher detection rate of pathogenic variants in WGS compared to WES and TCS in the coding and NCSS regions^2,20^. For instance, in seven of 24 solved cases (29.2%), one or both pathogenic alleles in the coding regions of *EYS* and *HGSNAT* were not detected or remained unrecognized as pathogenic in the previous genetic testing methods. This is due to incomplete gene panels (previous absence of *HGSNAT*), flaws in the annotation pipeline, or a lack of evidence or knowledge regarding the implication of these variants as IRD-associated at the time of WES or TCS data analysis. In one case (Pt-12), we have identified a missense variant in *EYS*, c.5044G>T, which was assigned previously as “variant with unknown significance”^34^. However, due the strong in silico predictions for this missense variant and the highly conserved amino acid and nucleotide, we categorized this patient as likely solved. We have identified the hypomorphic allele p.Ala615Thr in *HGSNAT* in two homozygous (Pt-15 and Pt-18) and three heterozygous (Pt-16, Pt-17, and Pt-19) patients. After extensive analysis of IRD- and ciliopathies-associated genes as well as the complete sequence of *HGSNAT*, no other candidate variant was identified in these probands. Recently, Schiff et al. investigated the enzymatic activity of the HGSNAT protein and showed the decreased enzyme activity and urinary GAG/creatinine ratio in patients homozygous for p.Ala615Thr. They suggested that this allele is associated with non-syndromic retinal disease with the influence of transacting genetic and/or environmental modifiers on the retina^35^. Currently, our cases have non-syndromic RP; however, we suggest monitoring as a protracted syndromic phenotype may arise.

In addition, WGS facilitated the discovery of pathogenic noncoding variants that were not previously captured by TCS and WES. Two NCSS variants, i.e. *HGSNAT*, c.493+5G>A and *USH2A*, c.4758+3A>G, were detected using WGS. These noncoding variants were overlooked in earlier testing methods due to the lack of evidence for pathogenicity at the time of sequencing. After in vitro splice assays of these variants, they were both classified as severe due to the presence of <25% of the wild-type fragment in the total mRNA^18^ (Fig. 2 and Supplementary Table 5).

In Pt-7, we identified *CDHR1* c.783G>A in a homozygous state, which previously was determined to lead to in-frame skipping of exon 8 (p.Asp214_Pro261del)^26^. No other potentially causative variants were identified as part of our analysis. The allele frequency of this variant (total allele frequency of 0.003052 and allele frequency of 0.004903 in non-Finnish Europeans; Supplementary Table 6) indicates that this variant may not be fully penetrant or acts as a hypomorphic allele. The splice defect is in-frame and may confer a subtle change in protein function. Additionally, Stingl et al. showed reduced expression of the mutant allele by comparison with the wild type^26^. In line with this, Pt-7 displays a mild phenotype consistent with previously described *CDHR1*-related disease and late age at disease onset^26^. It is also possible that other genetic factors affect the penetrance of this variant, potentially explaining the relatively high allele frequency^35^.

Importantly, WGS identified pathogenic coding variants in IRD-associated genes that did not reside in the TCS panels. Genes such as *FAM161A* (NG_028125.1), *RGS9BP* (NG_016751.1), *C21ORF2* (NG_032952.1), and *CDHR1* had not been included in the TCS panels at the time of sequencing (Table 2). These findings prompt redesign of TCS panels to include these genes where WES and WGS remain too costly to apply on a wider scale.

In seven out of 24 (29.2%) resolved cases, we identified five incidents of deep-intronic variants that could not be detected with previous sequencing techniques. The tested deep-intronic variants in this study led to frameshift variants, except for *GRM6*, c.1355-587dup, and were classified as severe alleles with <25% of remaining wild-type mRNA^18^. We observed the insertion of a 75-nt pseudoexon in *GRM6* midigene assays using the mutant construct (21%), which encodes 25 amino acids. In addition, a partial deletion of exon 6 in wild-type and mutant midigene assays was identified, which was more abundant in products from the wild-type midigene compared to those from the mutant midigene (54% compared to 37% in the mutant) (Fig. 3b). Of note, the partial exon 6 deletion in *GRM6* was not reported previously, and further research needs to be undertaken to investigate this phenomenon in a retina-specific cell model. The deletion of part of exon 30 in *ABCA4* has previously been reported in normal retinal mRNA^4,36^. In addition, we classified *GRM6* c.1355-587dup as a mild allele in this study, although a stronger effect on splicing in retina-specific cells cannot be discarded as a similar effect was previously observed for the *ABCA4* c.4539+2028C>T and c.5196+1137G>A variants. These findings highlight the importance of functional analyses in proving intronic variant pathogenicity, the contribution of such variants to IRD pathogenesis, and the diagnostic imperative imposed by them.

No splice defects were observed as a result of *ABCA4*, c.6148-89G>A (Pt-27); *ABCA4*, c.5460+1315_5460+1317delinsTA (Pt-28-30); *CYP4V2*, c.214+879_214+882delinsG (Pt-52); *PDE6B* c. 469-776C>G (Pt-61); and *RLBP1* c.525+425_525+433delinsATA (Pt-65). For the *ABCA4*, *CYP4V2*, and *RLBP1* indel variants, only Alamut prediction scores were used for their inclusion in the in vitro functional analysis as the SpliceAI algorithm cannot provide prediction scores for indel variants. All three variants create a cryptic donor site and have not been reported previously in the gnomAD database (Supplementary Fig. 2A, C, and E). The *ABCA4*, c.6148-89G>A variant strengthens the cryptic SDS in intron 44, from 67.6 to 79.1 in SpliceSiteFinder-like (range 0–100), and also activates an exonic splice enhancer (SF2/ASF) motif in the presence of c.6148-89G>A (Supplementary Fig. 2B). Similarly, the SpliceAI algorithm predicts a gain of a nearby SDS with a delta score of 0.12 (Supplementary Table 4). Surprisingly, we did not observe a splice defect in the presence of variant c.6148-89G>A in *ABCA4*. Possibly, the functional assay in HEK293T cells is not a representative model for this variant due to differences in the presence of splice enhancer and silencer proteins in kidney and retina-specific cells. These five variants and in particular the *ABCA4*, c.6148-89G>A variant, may still be proven pathogenic when investigated in retina-like cells. Such findings have been reported previously, e.g., splice defects resulting from *ABCA4* c.4539+2001G>A and c.4539+2028C>T were only detected in photoreceptor precursor cells derived from patient fibroblasts^4^. Therefore, for variants that do not display a splice defect, exploring variant pathogenicity in models that better recapitulate the retinal cell identity should be considered. Likewise, we have established that a minority of wild-type mRNA remains for a number of variant alleles, while analysis of variants in photoreceptor precursor cells may determine an alternative allele severity compared to the result from in vitro splice assays in HEK293T cells. Furthermore, Pt-52 was included as a suspected Bietti crystalline corneoretinal dystrophy case, which has a strong genotype–phenotype association to pathogenic variants in *CYP4V2*. This may support our hypothesis that providing pathogenic evidence for deep-intronic variants possibly failed due to the disadvantages of the HEK293T midigene system. Alternatively, the disadvantage lies within the flaws of short-read sequencing to detect the actual pathogenic variants or the initial pathogenic allele may represent a chance-finding.

WGS is the optimal sequencing method for the detection of SVs by comparison with WES and TCS, as indicated by previous studies^2,19,37^. In line with this, we have detected a homozygous 3.5 kb SV in *RPGRIP1* in an individual with LCA (Pt-22), which remained undetected by WES. The SV led to exon 20 deletion in the mRNA, altering the open-reading frame and resulting in a stop codon after six amino acids (p.Asp1080Glyfs*6) (Supplementary Fig. 1D). The assessment of WES was set to only detect deletions when spanning 2 exons or more and therefore could not detect this SV. Previously, heterozygous pathogenic variants in *CEP290*, *PDE6A*, and *RPE65* were reported as the potential first allele, with all three genes strongly associated with the given phenotype, which may have distracted from assessing other clinically relevant genes such as *RPGRIP1* (Supplementary Table 1). However, a comprehensive analysis of the entirety of these genes did not identify additional pathogenic variants. This finding emphasizes the importance of analysis of all IRD-associated genes to identify the true genetic cause of disease.

In addition, a 26.86 kb heterozygous deletion on chromosome 19 was detected in proband Pt-23 (Supplementary Fig. 2B). This deletion encompasses the entire *PRPF31* and *TFPT* genes, and the promoter of *NDUFA3*. This large deletion was not detected using TCS as the breakpoints of the variant lay within noncoding regions not covered by the TCS panel. In general, it is difficult to detect read depth reduction in heterozygous SVs when there is no read coverage in the surrounding region for comparison. In addition, this specific individual was thought to have an autosomal recessive disease given the lack of family history. Segregation analysis confirmed maternal inheritance of the SV. However, the proband’s mother was phenotypically unaffected, due to incomplete penetrance associated with variants in *PRPF31*, noted in other studies^38–40^. *TFPT* is a molecular partner of *TCF3* and is associated with childhood acute lymphoblastic leukemia (OMIM: 613065)^41^. *NDUFA3* is required for the formation of the extramembrane arm of human mitochondrial complex 1^42^. No additional disease features could be attributed to the disruption of these genes at this time. In addition, there are no reported pathogenic variants in either *TFPT* or *NDUFA3* in the ClinVar database (https://www.ncbi.nlm.nih.gov/clinvar/). A similar deletion encompassing *PRPF31*, *TFPT*, and *NDUFA3* on chromosome 19 has been reported previously^43^.

In this study, 76 (76%) cases remain genetically unresolved following WGS analysis, indicating the presence of pathogenic variants that eluded our data analysis approach. The pipeline employed in this study was designed to be stringent, comprehensive, and time effective, given the immense volume of data created by WGS. Unresolved cases may harbor pathogenic variants that were called, but not individually interpreted because they did not pass the prioritization criteria employed. Our data analysis approach relied on the presence of one coding or NCSS pathogenic variant before interrogation of intronic regions of the same gene. Therefore, it is possible that some unresolved probands carry homozygous or compound heterozygous pathogenic intronic variants. Alternatively, some may carry pathogenic variants in genes not yet associated with IRD and therefore were not included in the WGS data analysis.

Furthermore, it is likely that some individuals carry pathogenic variants located within regulatory regions of IRD genes, as reported in other studies^44,45^. However, the identification and interpretation of such variants remains complex. These variants are possibly not prioritized by our current strategy as they likely have their own interpretation criteria that are yet to be established. In addition, it is important to note that the line between Mendelian and complex disease may be blurred by modifier loci and environmental influences^46^. Thus, we should consider that some of the remaining unresolved IRD cases in this study may have a more complex etiology than previously thought as shown by the presence of hypomorphic alleles in cases of our cohort.

Lastly, it is possible that some variants may not have been called by variant calling software as they may be located in repetitive or low complexity regions with poor coverage, e.g., *RPGR* open-reading frame 15 (*ORF15*). Pathogenic variants in *RPGR* *ORF15* are responsible for 50–90% of X-linked RP (MIM: 312610)^47^. While in many instances male cases are analyzed for pathogenic variants in *RPGR* *ORF15* prior to WES, in our WGS cohort, 35/76 (46%) of unresolved cases are male individuals with an RP phenotype and it is possible that a proportion carries pathogenic variants in *RPGR ORF15*. As data analysis tools for WGS and knowledge surrounding variant interpretation improve, it is anticipated that further cases will receive a genetic diagnosis in the future.

We performed WGS on a cohort of 100 IRD probands that remained unresolved following the employment of TCS and WES as preliminary sequencing measures. A genetic cause of the disease was determined in 24 (24%) cases through detection of variants in genes that were not included in TCS panels or by identification and functional analyses of pathogenic intronic variants as well as detection of both small and large SVs not previously identified or recognized as causative. While a 24% yield may be modest, the number of “obvious” genetic causes detected in the coding regions of established IRD-associated genes is surprising, especially, considering the extensively previously examined cohort we assessed. Reasons for this may include an incomplete gene panel, poor read quality in previous testing methods, or lack of evidence surrounding the implication of some IRD genes at the time of initial sequencing. While the coverage and quality of reads in WGS are higher than prescreening techniques enabling better variant detection in this cohort, our findings emphasize the necessity of improvements in variant interpretation and bioinformatics pipelines as well as performing in vitro functional assays to obtain a higher diagnostic yield not only by WGS but also by less comprehensive sequencing techniques such as WES and TCS.

The findings of this study demonstrate that WGS can be an optimal sequencing approach for individuals diagnosed with an IRD. In future studies, a combined approach of WGS and optical genome mapping or long-read sequencing can be beneficial to overcome the difficulties of SV detection in short-read sequencing and provide a higher genetic diagnostic yield. It is a diagnostic imperative to continue to analyze individuals who have received an inconclusive genetic testing result following less comprehensive sequencing methods. Identification of the underlying genetic pathogenesis of a disease is becoming increasingly important in the advent of gene-based therapeutics, where a known genetic cause is essential to access clinical trials and approved treatments. With decreasing costs, increasing data interpretability, and continued functional analysis, we foresee that WGS may become the sequencing methodology of choice to provide IRD patients with an accurate genetic diagnosis.

## Methods

### Ethical considerations

The study adhered to the tenets of the Declaration of Helsinki and was approved by the local ethics committees of the Radboud University Medical Center (Nijmegen, The Netherlands), the Rotterdam Eye Hospital (Rotterdam, The Netherlands) (MEC-2010-359; OZR protocol no. 2009-32), The Department of Ophthalmology, The Royal Victoria Eye and Ear Hospital (Dublin, Ireland) (13-06-2011: HRA-POR201097), Ramabam Health Care Campus (Haifa, Israel), and HaEmek Medical Center (Afula, Israel). Written informed consent was obtained from patients prior to DNA analysis and inclusion in this study.

### Patient cohort

100 IRD probands were subjected to WGS, of which 43 were recruited from the Radboud University Medical Center, Nijmegen and The Rotterdam Eye Hospital (both in The Netherlands), 19 from the Technion-Israel Institute of Technology (Haifa, Israel), and 38 from the Research Foundation of the Royal Victoria Eye and Ear Hospital (Dublin, Ireland). A suspected recessive inheritance pattern and unresolved status following prior preliminary genetic testing using TCS or WES were prerequisites for participation in this study.

### DNA acquisition and preliminary genetic analysis

Participants provided either a blood or saliva sample for analysis. DNA from Irish participants was isolated using the Qiagen DNA Blood Maxi Kit (Hilden, Germany) or the DNA Genotek Oragene-DNA Kit (Ontario, Canada). In the remaining individuals, the genomic DNA was extracted according to the standard protocol^48^. TCS and subsequent data analysis of single-nucleotide variants (SNVs), SVs, and CNVs were carried out as described previously in the Irish cohort^6,9^ and eight Israeli cases^49,50^ (Supplementary Note 1). All Dutch (43 cases) and remaining Israeli (11 cases) participants had undergone WES (Supplementary Note 1) and the subsequent data were analyzed based on the identification of known pathogenic variants as well as likely pathogenic variants or variants of unknown significance, i.e., assessment of all type of variant including SNVs, SVs, and CNVs. Variant prioritization in WES data was based on a strong correlation between genotype and phenotype in individuals, i.e., a first pathogenic allele was identified in a gene previously found to be mutated in the IRD cases with a similar phenotype. Thirty-three out of 43 Dutch, 14 out of 38 Irish, and nine out of 19 Israeli cases were monoallelic, carrying one reported pathogenic or candidate allele in IRD-associated genes prior to WGS.

WGS was performed by BGI (Hongkong, China) on a BGISeq500 using either 2 × 100 or 2 × 150 bp paired-end reads, with a 30-fold minimal median coverage per genome. Burrows–Wheeler Aligner was utilized to map the data to the human genome (hg19). The quality of the WGS data was based on the insert size, percentage mapped reads, percentage duplicated mapped reads, coverage, bases with >20× coverage and error rate, which were evaluated using Qualimap V.2.2.1^51^. Variant calling was performed by xAtlas V.0.1^52^ and variants were annotated using the Variant Effect Predictor (VEP V.91) and Gencode V.34lift37 basic gene annotations. It consists of various features such as chromosomal and nucleotide position, gene name, and its component (e.g., intron, exon, splice site, 5′-untranslated region (5′-UTR), 3′-UTR, intragenic). In addition, we used an in-house developed pipeline that provides further information including different population frequency databases (e.g., gnomAD^22^ (https://gnomad.broadinstitute.org), GoNL^53^, Wellderly^54^, 1000genomes^55^, in-house variant frequencies database containing WES data of 15,576 individuals), various in silico prediction scores (e.g., Grantham, PhyloP, CADD_PHRED, SpliceAI)^56–59^, predicted protein effect, gene and disease OMIM description, and gene regulation and expression data. Runs of homozygosity were detected using plink V.1.07^60^ with the following parameters: homozyg-window-het=3, homozyg-snp=50 and homozyg-kb=300.

SVs were detected based on paired-end and split-read evidence using Manta Structural Variant Caller V.1.1.0 (Illumina) with default parameters^61^. CNVs were called using Control-FREEC, which detects copy number changes and allelic imbalances based on the read depth^62^. SVs and CNVs were annotated using an in-house developed pipeline^63^, i.e., annotations for chromosomal location and position, gene(s) and their component (e.g., intronic, exonic), type of SV (gain/loss), percentage overlap and frequency of various population frequency databases (e.g., gnomAD-SV^22^, GoNL^53^, Decipher^64^, Wellderly^54^, 1000genomes^55^), and disease OMIM description. Short tandem repeats (STRs) for 33 known pathogenic sites were detected using Expansion Hunter V.3.1.2. using default settings^65^.

### Variant prioritization approach

Sequencing data from a customized panel of 446 IRD and ocular defect-associated genes and 163 ciliopathy associated genes were extracted from the WGS data obtained from each patient. To facilitate the identification of pathogenic variants, a two-step protocol was designed for variant prioritization, in which the first step was the utilization of an automated prioritization pipeline composed in R studio^66^. The details of this pipeline can be found in Supplementary Note 2. The second step was a manual review of the remaining variants identified as follows below.

The manual variant prioritization approach differed depending on the findings of previous genetic studies. In cases where no first candidate variant was identified during previous genetic testing efforts, CNVs and SVs were evaluated first followed by assessment of SNVs. Subsequently, SNVs were manually prioritized according to their predicted pathogenic effect (i.e., nonsense, frameshift, canonical splice site variants, NCSS variants, in-frame deletions/insertions, missense variants, and synonymous variants). Coding or noncoding SNVs with a minor allele frequency of >1% in the gnomAD database were not considered causative^22^. Once a candidate pathogenic variant was identified, the predicted effects of rare intronic variants (with allele frequency <1%) in the gene of interest were interrogated in silico.

For cases where a first candidate variant in an IRD or ciliopathy associated gene was previously identified, the assessment of CNVs, SVs, and subsequently all rare intronic SNVs in the gene of interest was performed to identify a second causal variant. If a second causal variant remained elusive, an analysis of all CNVs, SVs, and SNVs was implemented in the same manner as for cases in which no first candidate variant was previously established. In addition, all IRD cases were analyzed for putative pathogenic STR expansions in IRD-associated genes.

### Variant frequency and pathogenicity prediction parameters

All CNVs and SVs were assessed based on their potential effects to disrupt the reading frame or regulatory regions in 5′- and 3′-UTRs. SVs were compared to previously identified SVs reported in gnomAD-SV and the Database of Genomic Variants. In the presence of pathogenic SVs, the breakpoint regions were manually assessed for the presence of microhomology or repetitive elements.

For the coding SNVs, the pathogenicity of missense variants was measured using threshold scores of in silico prediction tools; PhyloP (range 14.1–6.4; predicted pathogenic ≥2.7)^56^, CADD-PHRED (range 1–99; predicted pathogenic ≥15)^57^, and Grantham (range 0–215; predicted pathogenic ≥80)^58^. Missense variants that only passed one of the thresholds were not considered causative.

Noncoding variants were selected for in vitro splice assay analysis based on criteria that were defined previously^18^. In short, the predicted effect of noncoding variants on splicing was evaluated using the algorithms SpliceSiteFinder‐like, MaxEntScan, GeneSplicer, and Human Splicing Finder embedded in the Alamut Visual software version 2.10 (Interactive Biosoftware, Rouen, France; http://www.interactive-biosoftware.com)^67–71^. In addition, the SpliceAI algorithm^59^ was utilized to select candidate noncoding variants. Other than the default for SpliceAI, we selected variants with at least one delta score above the threshold of 0.02 for SDS or SAS gain or loss. For each variant, 500 bp upstream and downstream were included as the input sequences for SpliceAI analysis.

### In vitro splice assays

After completing the data analysis of 100 cases, 10 deep-intronic and 3 NCSS candidate variants in 14 individuals were identified, which adhered to the stringent criteria defined above for noncoding variants. Subsequently, a midigene or minigene splice assay was employed as described previously to assess potential splicing defects in the presence of variants^63,72^. In short, the regions of interest of a genomic DNA sample were amplified by primers that contain attB1 and attB2 tags at their 5′ end to facilitate the Gateway cloning. After generating the wild-type constructs, they served as templates to generate mutant constructs by PCR-based site-directed mutagenesis. The *ABCA4* variant c.6148-89G>A was introduced into a vector containing the wild-type fragment of *ABCA4* from exons 43 to 47, as described previously^15^. For *PDE6B* c. 469-776C>G in Pt-61 and *RLBP1* c.525+425_525+433delinsATA in Pt-65, mutant and wild-type constructs were both generated from the patient’s DNA as the mutagenesis PCR technique failed due to the complexity of the regions surrounding these variants. Subsequently, the wild-type and mutant constructs were separately incorporated into the pCI‐*NEO‐RHO* or pcDNA3native/DEST Gateway‐adapted vector to generate wild-type and mutant midi(mini)genes. The pcDNA3native/DEST vector was utilized when the region of interest contained the first exon of the gene. Generated wild-type and mutant midigenes and minigenes were independently transfected into HEK293T cells. After 48 h of incubation, mRNAs were isolated and utilized for transcript analysis by RT-PCR with primers in flanking exons for midigenes or primers in *RHO* exon 3 and 5 for minigenes. All primers designed for splice assays are listed in Supplementary Table 7. Electrophoresis gel images are derived from raw images that are provided in Supplementary Fig. 3. The Fiji software was utilized for quantification analysis in the presence of multiple mRNA fragments after gel electrophoresis. The variants with <25% of the wild-type fragments present in mutant constructs were classified as severe alleles. This variant classification refers only to the severity of the mRNA defect observed in HEK293T cells.

### Reporting summary

Further information on research design is available in the Nature Research Reporting Summary linked to this article.

## Supplementary information

Reporting Summary

Supplementary Information

## Data Availability

All data relevant to the study are included in the article or uploaded as Online Supplementary data. The pathogenic variant data are submitted to Leiden Open Variation Database (LOVD). The whole-genome sequencing data are not publicly available as these could compromise research participant privacy. Whole-genome sequencing data may become available upon a data transfer agreement approved by local (Irish, Israeli and/or Dutch) ethical committees. Patient sample identifiers from this study can be released upon reasonable request from “Pt-1 to Pt-100” to the corresponding local “DNA-number.” Specific variant requests or other data are available from the corresponding author (S.R.) upon reasonable request.
