# Supplementary material for: Whole genome sequencing and in vitro splice assays reveal genetic causes for inherited retinal diseases
Source: NPJ Genom Med. 2021 Nov 18;6:97. doi: 10.1038/s41525-021-00261-1 (PMC8602293; doi:10.1038/s41525-021-00261-1)
Supplement: Supplementary file 1 — Reporting Summary [file 41525_2021_261_MOESM1_ESM.pdf]

nature portfolio

Corresponding author(s): NPJGENMED-00992
Last updated by author(s): 2021-8-9

Reporting Summary

Nature Portfolio wishes to improve the reproducibility of the work that we publish. This form provides structure for consistency and transparency in reporting. For further information on Nature Portfolio policies, see our Editorial Policies and the Editorial Policy Checklist.

Statistics

For all statistical analyses, confirm that the following items are present in the figure legend, table legend, main text, or Methods section.

n/a	Confirmed	
☐	☒	The exact sample size (*n*) for each experimental group/condition, given as a discrete number and unit of measurement
☐	☒	A statement on whether measurements were taken from distinct samples or whether the same sample was measured repeatedly
☒	☐	The statistical test(s) used AND whether they are one- or two-sided<br>*Only common tests should be described solely by name; describe more complex techniques in the Methods section.*
☒	☐	A description of all covariates tested
☒	☐	A description of any assumptions or corrections, such as tests of normality and adjustment for multiple comparisons
☒	☐	A full description of the statistical parameters including central tendency (e.g. means) or other basic estimates (e.g. regression coefficient) AND variation (e.g. standard deviation) or associated estimates of uncertainty (e.g. confidence intervals)
☒	☐	For null hypothesis testing, the test statistic (e.g. *F*, *t*, *r*) with confidence intervals, effect sizes, degrees of freedom and *P* value noted<br>*Give P values as exact values whenever suitable.*
☒	☐	For Bayesian analysis, information on the choice of priors and Markov chain Monte Carlo settings
☒	☐	For hierarchical and complex designs, identification of the appropriate level for tests and full reporting of outcomes
☒	☐	Estimates of effect sizes (e.g. Cohen's *d*, Pearson's *r*), indicating how they were calculated

Our web collection on statistics for biologists contains articles on many of the points above.

Software and code

Policy information about availability of computer code

Data collection	*Provide a description of all commercial, open source and custom code used to collect the data in this study, specifying the version used OR state that no software was used.*
Data analysis	The whole genome sequencing data were processed as described in the methods. Variant prioritization has been performed through a automated prioritisation pipeline provided in Supplemental File 2. The online available algorithms SpliceSiteFinder-like, MaxEntScan, GeneSplicer, and Human Splicing Finder embedded in the Alamut Visual software version 2.10 (Interactive Biosoftware, Rouen, France; http://www.interactive-biosoftware.com.) were used. Moreover, the online available the SpliceAI algorithm was employed with adjusted settings as described in the methods. Candidate variants and their respective RNA and protein annotation have been submitted to the Leiden open (source) variation databases (LOVD) (www.lovd.nl)

For manuscripts utilizing custom algorithms or software that are central to the research but not yet described in published literature, software must be made available to editors and reviewers. We strongly encourage code deposition in a community repository (e.g. GitHub). See the Nature Portfolio guidelines for submitting code & software for further information.

Data

Policy information about availability of data

All manuscripts must include a data availability statement. This statement should provide the following information, where applicable:

- Accession codes, unique identifiers, or web links for publicly available datasets
- A description of any restrictions on data availability
- For clinical datasets or third party data, please ensure that the statement adheres to our policy

The data that support the findings of this study are available on request from the corresponding author [SR]. The data are not publicly available as these could compromise research participant privacy.

Field-specific reporting

Please select the one below that is the best fit for your research. If you are not sure, read the appropriate sections before making your selection.

☒ Life sciences ☐ Behavioural & social sciences ☐ Ecological, evolutionary & environmental sciences

For a reference copy of the document with all sections, see nature.com/documents/nr-reporting-summary-flat.pdf

Life sciences study design

All studies must disclose on these points even when the disclosure is negative.

Sample size	A cohort of 100 genetically unexplained cases was included for a whole genome sequencing study.
Data exclusions	No data was excluded
Replication	No replications have been performed
Randomization	No randomisation was performed
Blinding	Personalia were blinded to the researchers apart for the lead investigator to communicate with the respective clinicians about potential genetic findings. No other blinding is relevant to the study.

Reporting for specific materials, systems and methods

We require information from authors about some types of materials, experimental systems and methods used in many studies. Here, indicate whether each material, system or method listed is relevant to your study. If you are not sure if a list item applies to your research, read the appropriate section before selecting a response.

Materials & experimental systems

n/a	Involved in the study	
☒	☐	Antibodies
☐	☒	Eukaryotic cell lines
☒	☐	Palaeontology and archaeology
☒	☐	Animals and other organisms
☐	☒	Human research participants
☒	☐	Clinical data
☒	☐	Dual use research of concern

Methods

n/a	Involved in the study	
☒	☐	ChIP-seq
☒	☐	Flow cytometry
☒	☐	MRI-based neuroimaging

Eukaryotic cell lines

Policy information about cell lines

Cell line source(s)	HEK293T
Authentication	Purchased from ATTC. For more information: https://www.atcc.org/products/crl-3216
Mycoplasma contamination	All cultures are bi-weekly analyzed for mycoplasma contamination. Our cultures were negative.
Commonly misidentified lines (See ICLAC register)	none

March 2021

Human research participants

Policy information about studies involving human research participants

Population characteristics	Our study includes 100 cases that remained genetically unexplained in previous genetic testing. Cases origin from Ireland, Israel and the Netherlands.
Recruitment	Our study includes 100 cases that remained genetically unexplained in previous genetic testing.
Ethics oversight	The study adhered to the tenets of the Declaration of Helsinki and was approved by the local ethics committees of the Radboud University Medical Center, Nijmegen, The Netherlands, the Rotterdam Eye Hospital, Rotterdam, The Netherlands (MEC-2010-359; OZR protocol nr. 2009-32; Radboud protocol nr. 2018-4516), The Research Foundation, The Royal Victoria Eye and Ear Hospital, Dublin, Ireland (13-06-2011: HRA-POR201097), Ramabam Health Care Campus, Haifa, Israel and HaEmek Medical Center, Afula, Israel. Written informed consent was obtained from patients prior to DNA analysis and inclusion in this study.

Note that full information on the approval of the study protocol must also be provided in the manuscript.
