# Supplementary material for: Whole genome sequencing and in vitro splice assays reveal genetic causes for inherited retinal diseases
Source: NPJ Genom Med. 2021 Nov 18;6:97. doi: 10.1038/s41525-021-00261-1 (PMC8602293; doi:10.1038/s41525-021-00261-1)
Supplement: Supplementary file 2 — Supplementary Information [file 41525_2021_261_MOESM2_ESM.pdf]

Supplemental File

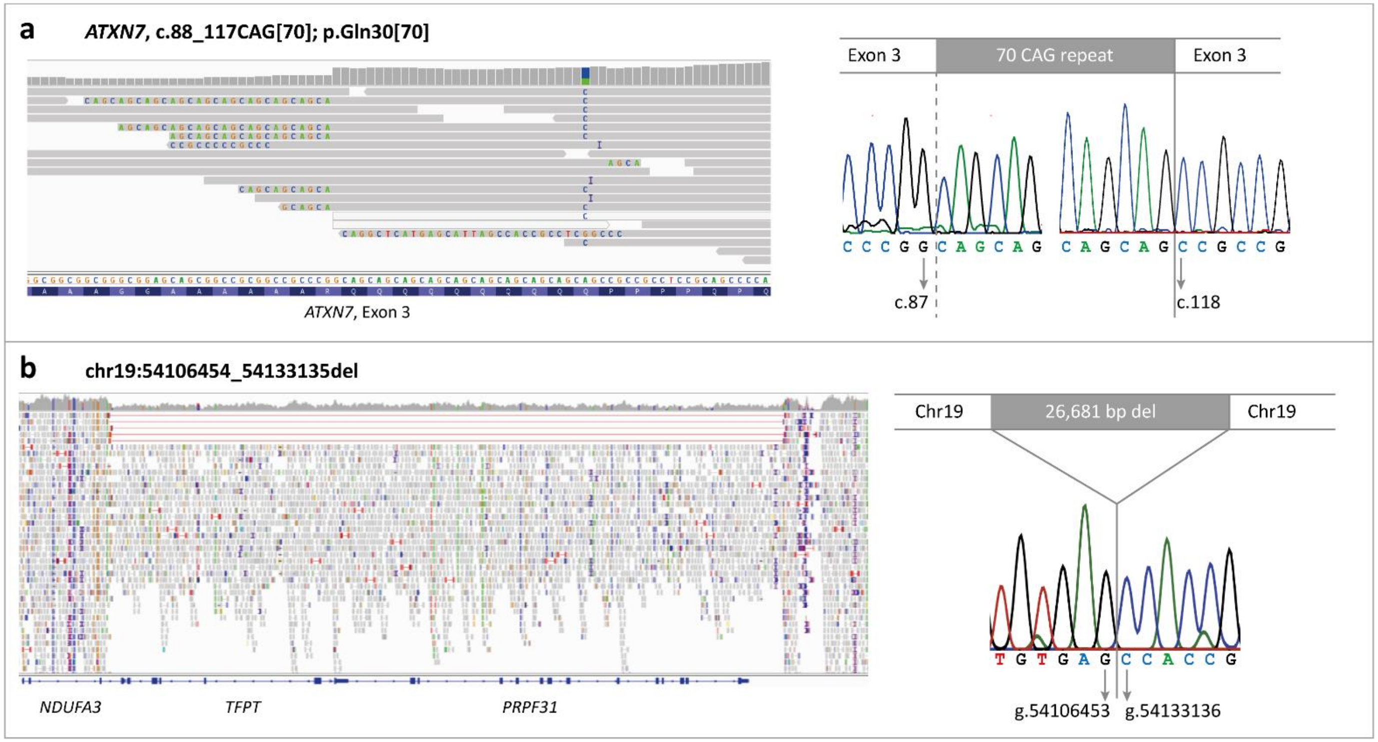

Supplementary Figure 1: Schematic representation of IGV software for pathogenic structural variants in this study. a) The CAG expansion repeat in *ATXN7* exon 3 is depicted in soft-clipped reads. We observed 70 repeats in the mutant allele, while the normal allele does not exceed 19 repeats. **b)** An ~26-kb pathogenic deletion on chromosome 19 detected by Control-FREEC, found in one proband is shown. Red lines across the region represent reads where the inferred insert size is larger than expected, indicative of a deletion. Similarly, the depth of coverage above the reads appears to decrease by approximately half by comparison with the surrounding sequences, pointing towards a heterozygous deletion. A cross-deletion PCR was performed that yielded a 700-bp fragment, which was subsequently Sanger sequenced (right) to confirm the breakpoints at the nucleotide level. The deletion encompasses *PRPF31, TFPT,* and the promoter of *NDUFA3.*

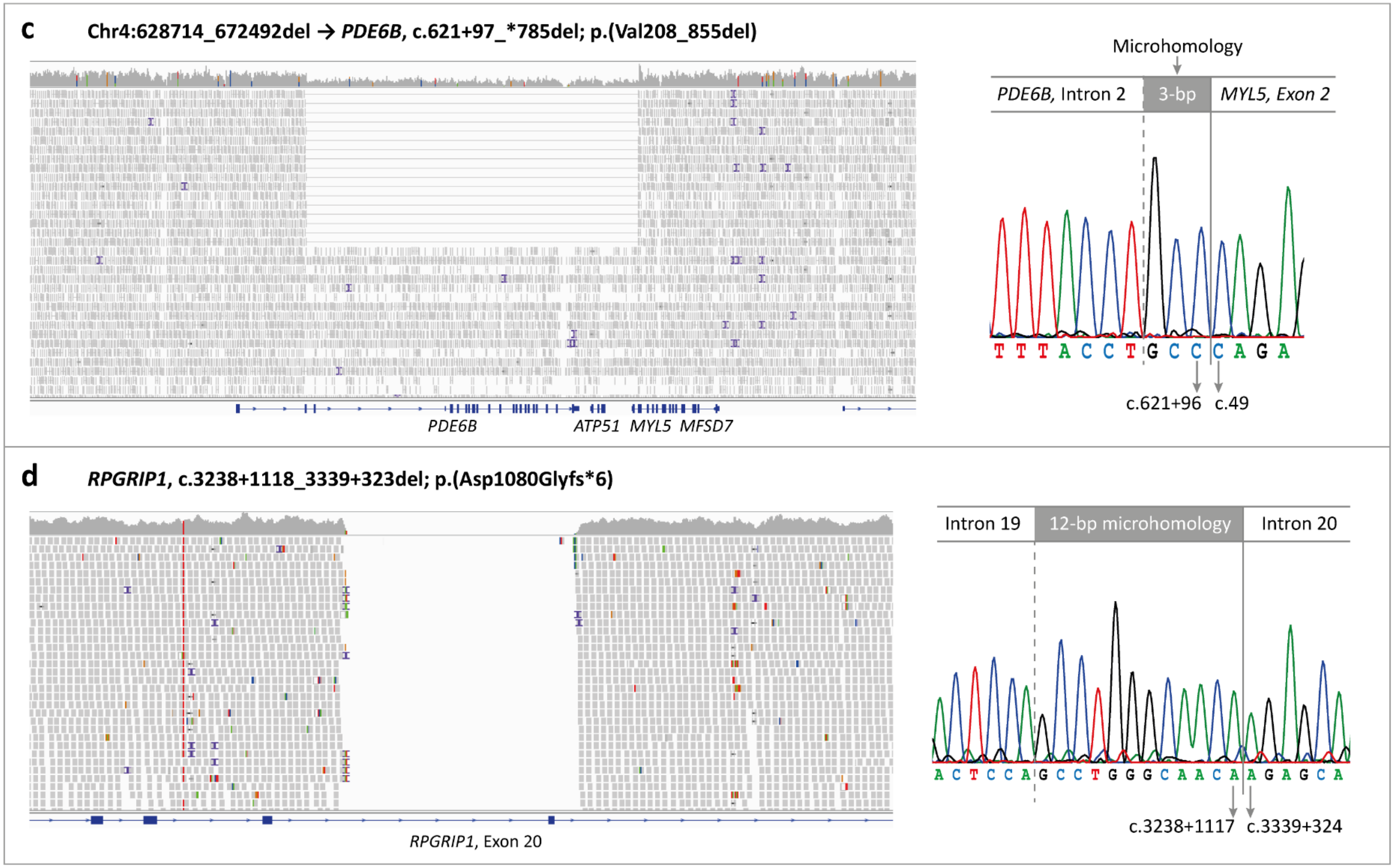

***Continued* Supplementary Figure 1: c)** The heterozygous deletion in *PDE6B* was detected by Control-FREEC tool in this affected individual. The gray lines across the region represent reads where the inferred insert size is larger than expected, indicative of a deletion. The Sanger sequence analysis confirmed the deletion at the nucleotide level. **d)** We identified the homozygous deletion in the proband which led to the deletion of exon 20 of *RPGRIP1*. The SV breakpoints were confirmed by Sanger sequencing shown in the right part. In *PDE6B* and *RPGRIP1* SVs, the 3-bp and 12-bp microhomologies at the breakpoints were indicated in the grey boxes.

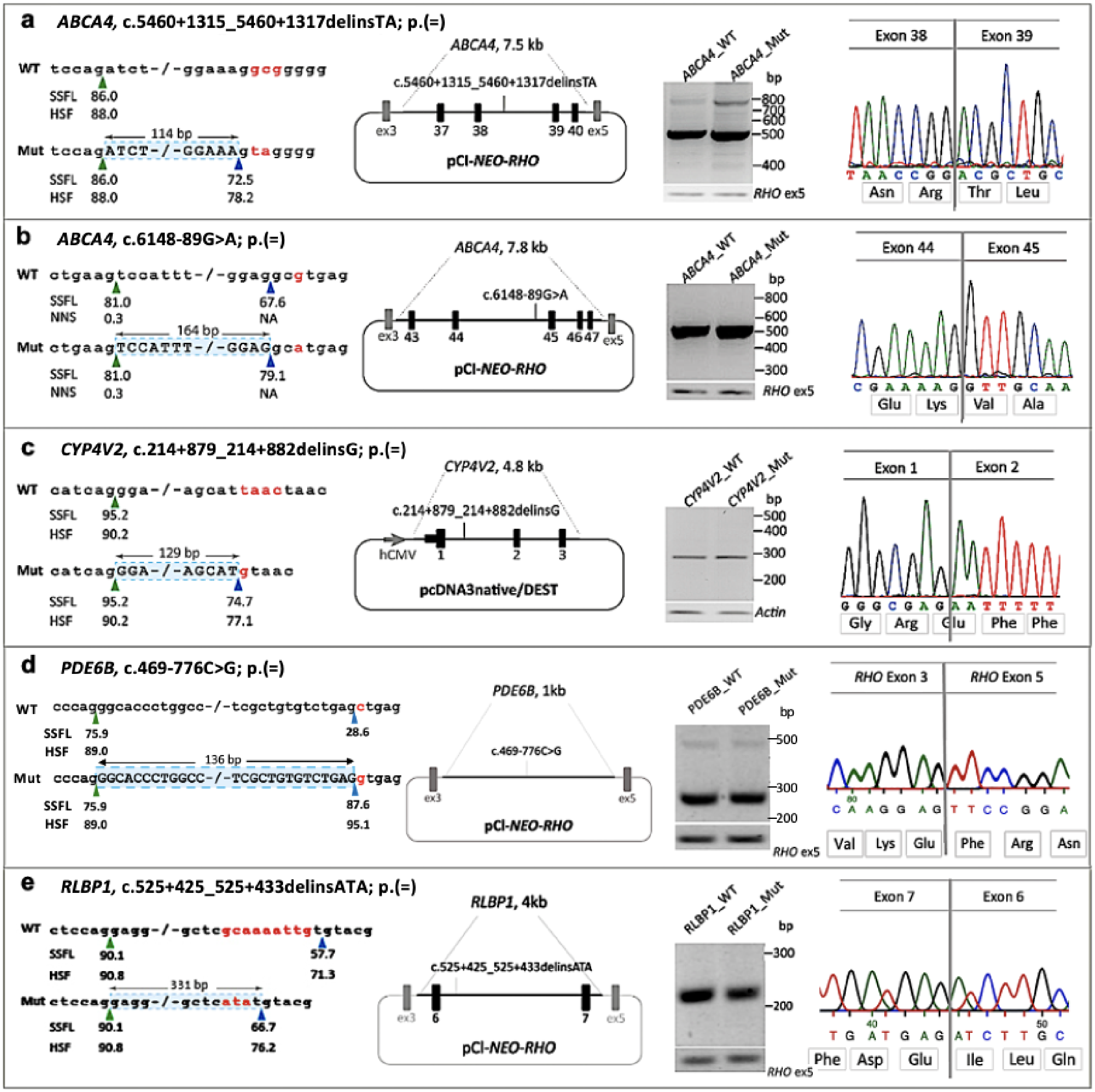

Supplementary Figure 2: Molecular genetic characterization of non-coding variants with no splice defects. For each variant, i.e. *ABCA4* 5460+1315_5460+1317delinsTA (**a**), *ABCA4*, c.6148-89G>A (**b**) *CYP4V2* c.214+879_214+882delinsG (**c**) *PDE6B* c.469-776C>G **(d)** and *RLBP1* c.525+425_525+433delinsATA **(e)** the left panels show the schematic representation of the gene of interest in the position of the corresponding variant in wild-type (WT) and in the presence of the variant (Mut). The SpliceSiteFinder-like (SSFL, range 0–100), NNSPLICE (NNS, range 0-1), and Human Splicing Finder (HSF, range 0–100) scores for the splice sites are indicated below each sequence. The green and blue triangles indicate the position of splice acceptor sites and splice donor sites in both wild-type and mutant sequences. The red highlighted nucleotides indicate the variants in the mutant sequence. The second panels showed the schematic representation of the mutant pCI-*NEO-RHO*.

They contain the variants and their nearby exons flanked by *RHO* exon 3 and 5 which was used to transfect HEK293T cells with wild-type and mutant constructs, respectively. For the *CYP4V2* c.214+879_214+882delinsG variant, the pcDNA3native/DEST vector with humanized CMV promoter was utilized due to the presence of the first exon in the construct. The next panels demonstrated the gel image of RT-PCR products which indicated no differences in wild-type and mutant midigenes. RT-PCR analysis of *RHO* exon 5 was performed as a control for transfection efficiency. In the last panels, the Sanger sequence analysis of the RT-PCR fragments confirmed no splice defect in each variant. For each variant, both WT and mutant variants were examined in the same experiment and processed in parallel.

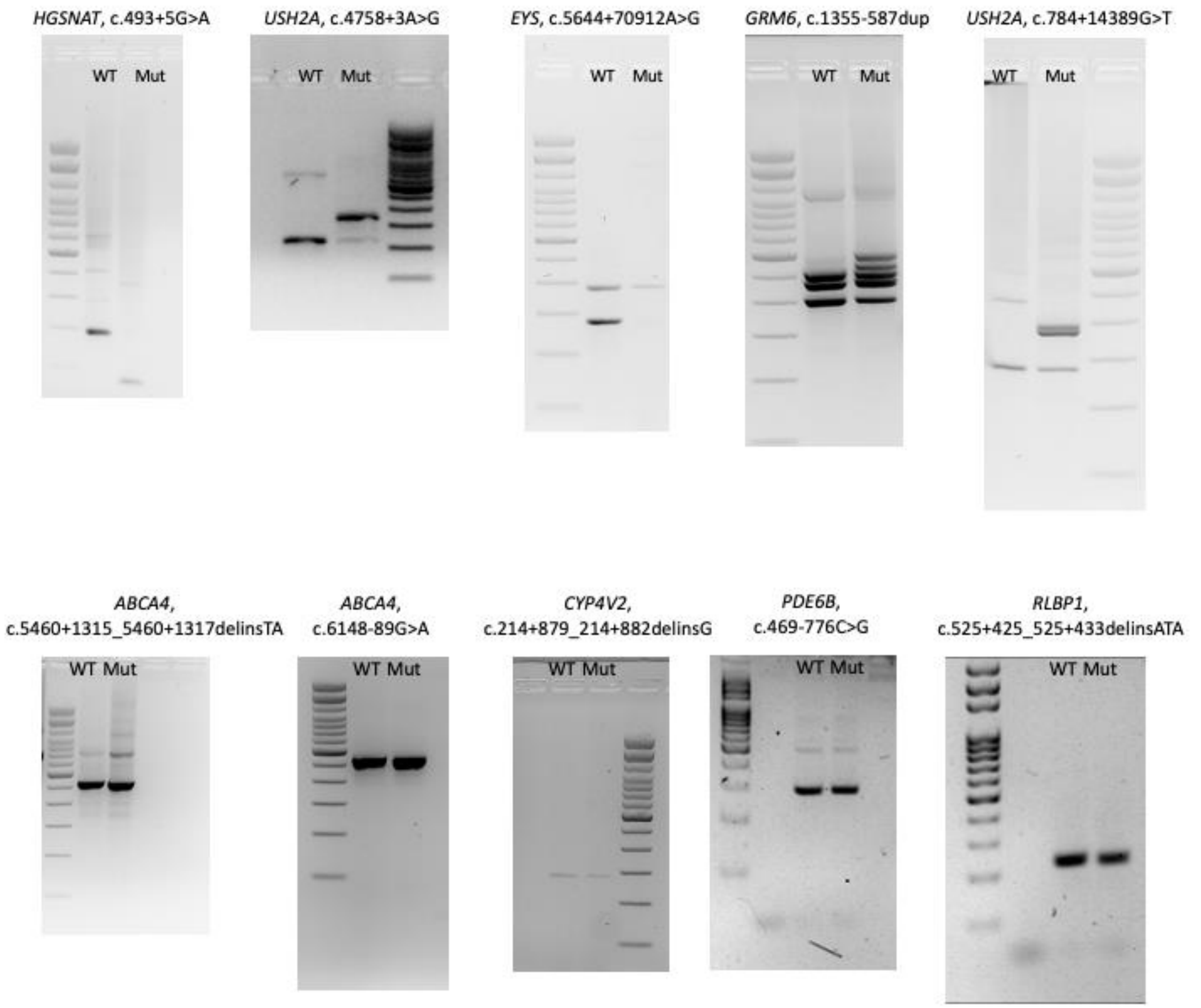

Supplementary Figure 3: Raw electrophoresis gel images obtained in this study. Raw electrophoresis gel images for each variant in order as shown in Figure 2, Figure 3 and Supplementary Figure 2.

Supplementary Table 1: Overview of candidate causative variants as first pathogenic allele identified prior to this study

ID	Phenotype	Gene	DNA allele 1	Protein allele 1	Previous genetic testing method	Causal gene identified in this study
**Solved cases**						
**Pt-1**	STGD1	*ABCA4*	c.6079C>T	p.(Leu2027Phe)	TCS	*ABCA4*
**Pt-2**	STGD1	*ABCA4*	c.6743T>C	p.(Phe2248Ser)	TCS	*ABCA4*
**Pt-3**	STGD1	*ABCA4*	c.5603A>T	p.(Asn1868Ile)	TCS	*ABCA4*
**Pt-4**	CRD*	n.r.	n.r.	n.r.	WES	*ATXN7*
**Pt-5**	RP	n.r.	n.r.	n.r.	TCS	*C21ORF2*
**Pt-6**	RP	n.r.	n.r.	n.r.	TCS	*C21ORF2*
**Pt-7**	RP	n.r.	n.r.	n.r.	TCS	*CDHR1*
**Pt-8**	AS	*ALMS1*	c.11997C>T	p.(Arg4003Trp)	TCS	***CEP78***
**Pt-9**	RP	*EYS*	c.[3906C>A;9468T>A]	p.[His1302Gln,Tyr3156*]	WES	*EYS*
**Pt-10**	RP	*EYS*	c.403_423delinsCTTTT	p.(Thr135Argfs*26)	TCS	*EYS*
**Pt-11**	CRD	n.r.	n.r.	n.r.	WES	*EYS*
**Pt-12**	RCD	*EYS*	c.1660T>A	p.(Cys554Ser)	WES	*EYS*
**Pt-13**	RP	n.r.	n.r.	n.r.	TCS	*FAM161A*
**Pt-14**	Oguchi disease	*GRM6*	c.1732C>T	p.(Arg578Cys)	WES	*GRM6*
**Pt-15**	RP	n.r.	n.r.	n.r.	WES	*HGSNAT*
**Pt-16**	RP	n.r.	n.r.	n.r.	WES	*HGSNAT*
**Pt-17**	RP	*HGSNAT*	c.1843G>A	p.Ala615Thr	WES	*HGSNAT*
**Pt-18**	RP	n.r.	n.r.	n.r.	WES	*HGSNAT*
**Pt-19**	RP	n.r.	n.r.	n.r.	TCS	*HGSNAT*
**Pt-20**	RP	*PCARE*	c.3604C>T	p.(Arg1202*)	TCS	*PCARE*
**Pt-21**	CRD	n.r.	n.r.	n.r.	WES	*RGS9BP*
**Pt-22**	LCA	*CEP290*	c.1079G>A	p.(Arg360Gln)	WES	***RPGRIP1L***
		PDE6A	c.2053G>A	p.(Val685Met)		
		RPE65	c.676G>A	p.(Val226Ile)		
**Pt-23**	RP	n.r.	n.r.	n.r.	TCS	*PRPF31*
**Pt-24**	RP	*PCARE*	c.2756_2768del	p.(Lys919Thrfs*2)	TCS	***USH2A***
**Unsolved cases**						
**Pt-25**	STGD1	*ABCA4*	c.6416G>C	p.(Arg2139Pro)	TCS	
**Pt-26**	MD	*ABCA4*	c.5882G>A	p.(Gly1961Glu)	WES	
**Pt-27**	STGD1	*ABCA4*	c.161G>A	p.[Cys54Serfs*14,Cys54Tyr]	TCS	
**Pt-28**	RP	*ABCA4*	c.[3758C>T;5882G>A]	p.[Thr1253Met,Gly1961Glu]	WES	
		EYS	c.2137+1G>A	p.(?)		
**Pt-29**	RCD	*ABCA4*	c.2701A>G	p.(Thr901Ala)	WES	
		IMPG2	c.3038C>T	p.(Pro1013Leu)		
**Pt-30**	CACD	*ABCA4*	c.5603A>T	p.(Asn1868Ile)	WES	

		IMPG2	c.745C>T	p.(Leu249Phe)		
**Pt-31**	RP	*ABCA4*	c.6148G>C	p.(Val2050Leu)	WES	
		PDE6B	c.655T>C	p.(Tyr219His)		
**Pt-32**	Nystagmus	*ALMS1*	c.8002C>T	p.(Arg2668*)	WES	
**Pt-33**	RP	*BBS1*	c.1169T>G	p.(Met390Arg)	TCS	
**Pt-34**	RP	*BBS2*	c.1033A>C	p.(Lys345Gln)	WES	
		CLUAP1	c.1093-2A>G	p.(?)		
**Pt-35**	RP	*BBS4*	c.1388G>C	p.(Gly463Ala)	TCS	
**Pt-36**	Clumped pigmentary retinal dystrophy	*BEST1*	c.404C>T	p.(Ala135Val)	WES	
**Pt-37**	MD	*BEST1*	c.1403C>T	p.(Pro468Leu)	WES	
**Pt-38**	RP	*CACNA2D4*	c.[1882C>T;2009-3C>T] [#]	p.[Arg628*,=]	TCS	
**Pt-39**	CD	*C21orf2*	c.862C>T	p.(Gln288*)	WES	
**Pt-40**	CRD	*CCDC138*	c.[1115C>T;1376G>A]	p.[Ser372Leu,Gly459Asp]	WES	
**Pt-41**	MD	*CEP290*	c.1079G>A	p.(Arg360Gln)	WES	
**Pt-42**	RP	*CEP290*	c.3176T>A	p.(Ile1059Lys)	WES	
**Pt-43**	CRD	*CEP290*	c.3812G>A	p.(Arg1271Gln)	WES	
		CLRN1	c.434-2A>T	p.(?)		
**Pt-44**	RP	*CNGA1*	c.959T>C	p.(Leu320Pro)	WES	
**Pt-45**	RP	*CNGB1*	c.2539G>A	p.(Gly847Arg)	TCS	
**Pt-46**	RCD	*CNGB1*	c.2747G>A	p.(Arg916His)	WES	
**Pt-47**	RP	*CNGB3*	c.1148del	p.(Thr383Ilefs*13)	WES	
**Pt-48**	MD	*CNGB3*	c.1208G>A	p.(Arg403Gln)	WES	
**Pt-49**	MD	*CNNM4*	c.502T>C	p.(Ser168Pro)	TCS	
**Pt-50**	RP	*CRB1*	c.135C>G	p.(Cys45Trp)	TCS	
**Pt-51**	USH-III	*CRB1*	c.664G>A	p.(Glu222Lys)	WES	
		USH1G	c.83C>T	p.(Pro28Leu)		
**Pt-52**	Bietti crystalline corneoretinal dystrophy	*CYP4V2*	c.610G>A	p.(Ala204Thr)	WES	
**Pt-53**	MD	*FAM161A*	c.1355_6delCA	p.(Thr452Serfs*3)	WES	
**Pt-54**	BBS	*FGD1*	c.935C>T	p.(Pro312Leu)	WES	
**Pt-55**	RP	*HGSNAT*	c.370A>T	p.(Arg124Trp)	TCS	
**Pt-56**	RP	*HSPA9*	c.1042C>T	p.(Arg348Cys)	WES	
**Pt-57**	RP	*IFT172*	c.406C>T	p.(Arg136Cys)	WES	
**Pt-58**	SLS	*IQCB1*	c.424_425del	p.(Phe142Profs*5)	WES	
**Pt-59**	LCA	*NMNAT1*	c.-57+13798_440-343dup	p.(Ala147Aspfs*8)	WES	
**Pt-60**	CRD	*PDE6B*	c.2363G>A	p.(Arg788His)	WES	
**Pt-61**	RP	*PDE6B*	c.621+97_*785del	p.(Val208_855del)	WES	
**Pt-62**	RP[$]	*PRPF31*	c.632G>A	p.(Arg211Gln)	WES	
**Pt-63**	CRD	*PRPH2*	c.133C>T	p.(Leu45Phe)	WES	
**Pt-64**	RP	*RBP3*	c.2153delC	p.(Pro718fs)	TCS	
**Pt-65**	RP	*RLBP1*	c.545T>G	p.(Phe182Cys)	WES	
**Pt-66**	RP	*SNRNP200*	c.4835C>T	p.(Thr1612Met)	WES	

**Pt-67**	CRD	*SNRNP200*	c.5644C>A	p.(Pro1882Thr)	WES	
**Pt-68**	CSNB	*TRPM1*	c.3121A>T	p.(Ile1041Phe)	TCS	
**Pt-69**	RP	*TULP1*	c.821delA	p.(Lys274Argfs*36)	TCS	
**Pt-70**	CD	n.r.	n.r.	n.r.	WES	
**Pt-71**	RP	n.r.	n.r.	n.r.	TCS	
**Pt-72**	CRD	n.r.	n.r.	n.r.	TCS	
**Pt-73**	RP	n.r.	n.r.	n.r.	TCS	
**Pt-74**	RP	n.r.	n.r.	n.r.	TCS	
**Pt-75**	RP	n.r.	n.r.	n.r.	TCS	
**Pt-76**	RP	n.r.	n.r.	n.r.	TCS	
**Pt-77**	RP	n.r.	n.r.	n.r.	TCS	
**Pt-78**	ACHM	n.r.	n.r.	n.r.	TCS	
**Pt-79**	CD	n.r.	n.r.	n.r.	TCS	
**Pt-80**	RP	n.r.	n.r.	n.r.	TCS	
**Pt-81**	RP	n.r.	n.r.	n.r.	TCS	
**Pt-82**	RP	n.r.	n.r.	n.r.	TCS	
**Pt-83**	STGD1	n.r.	n.r.	n.r.	TCS	
**Pt-84**	STGD1	n.r.	n.r.	n.r.	TCS	
**Pt-85**	RP	n.r.	n.r.	n.r.	TCS	
**Pt-86**	RP	n.r.	n.r.	n.r.	TCS	
**Pt-87**	RP	n.r.	n.r.	n.r.	TCS	
**Pt-88**	CRD	n.r.	n.r.	n.r.	TCS	
**Pt-89**	RP	n.r.	n.r.	n.r.	TCS	
**Pt-90**	RP	n.r.	n.r.	n.r.	WES	
**Pt-91**	RP	n.r.	n.r.	n.r.	WES	
**Pt-92**	FAP	n.r.	n.r.	n.r.	WES	
**Pt-93**	RP	n.r.	n.r.	n.r.	WES	
**Pt-94**	MD	n.r.	n.r.	n.r.	WES	
**Pt-95**	MD	n.r.	n.r.	n.r.	WES	
**Pt-96**	RP	n.r.	n.r.	n.r.	TCS	
**Pt-97**	RP	n.r.	n.r.	n.r.	TCS	
**Pt-98**	RP	n.r.	n.r.	n.r.	WES	
**Pt-99**	RP	n.r.	n.r.	n.r.	WES	
**Pt-100**	MD	n.r.	n.r.	n.r.	WES	

ACHM, achromatopsia; AS, Alström syndrome; CACD, central areolar choroidal dystrophy; CD, cone dystrophy; CRD, cone rod dystrophy; CSNB, congenital stationary night blindness; FAP, fundus albipunctatus; LCA, Leber congenital amaurosis; MD, macular dystrophy; RCD, rod cone dystrophy; RP, retinitis pigmentosa; SLS, Senior-Løken syndrome; STGD1, Stargardt disease; TCS, target capture sequencing; USH-III, Usher syndrome type 3; WES, whole exome sequencing. n.r., "not reported" indicates no candidate variant identified via previous methods. The bold font highlights an alternative causative gene identified from this study compared to the finding from previous genetic testing efforts. *CRD with neurodegeneration. $RP with hypogonadism. #Prior to segregation analysis that showed a *cis* configuration of the *CACNA2D4* c.[1882C>T;2009-3C>T] allele, the c.2009-3C>T variant was analyzed in a midigene splice assay as it has a total allele frequency of 0.0091 in gnomAD and a strong prediction to affect splicing. Using Alamut software, the predicted scores of the canonical splice acceptor site of exon 21 decreased, from 90.7 to 83.0

in Human Splicing Finder (range: 1-100), in the presence of the variant. Similarly, the SpliceAI algorithm predicts a loss of the SAS and SDS of exon 21 with a delta score of 0.12 and 0.10, respectively. In addition, exonic splice enhancer and silencer analysis indicate a loss in enhancer elements and a gain in silencer elements. The midigene splice assay for c.2009-3C>T did not show a splice defect (data not shown).

Supplementary Table 2: DNA and RNA notations for each variant found in 24 solved cases in this study

ID	Phenotype	Gene	g. position allele 1	DNA allele 1	RNA allele 1	gnomAD-AF	g. position allele 2	DNA allele 2	RNA allele 2	gnomAD-AF
**Pt-1**	STGD1	*ABCA4*	g.94471065G>A	c.6079C>T	r.6079c>u	0.0001980	g.94484001C>T	c.5196+1137G>A	r.[=, 5196_5197ins5196+1140_5196+1212]	0.00009558
**Pt-2**	STGD1	*ABCA4*	g.94461738A>G	c.6743T>C	r.6743u>c	0[#]	g.94492973G>A	c.4539+2028C>T	r.[=, 4539_4540ins4539+1891_4540-2162]	0.00006371
**Pt-3**	STGD1	*ABCA4*	g.94476467T>A	c.5603A>T	r.5603a>t	0.04219	g.94577010T>G	c.286A>C	c.286a>c	0.000003983
**Pt-4**	CRD*	*ATXN7*	g.63898362_63898391CAG[70]	c.88_117CAG[43]	r.(88_117cag[70])	0	_	_	_	n.a.
**Pt-5**	RP	*C21ORF2*	g.45753071C>G	c.218G>C	r.218g>c	0.0003255	g.45759002A>G	c.76T>C	r.76u>c	0
**Pt-6**	RP	*C21ORF2*	g.45753071C>G	c.218G>C	r.218g>c	0.0003255	g.45753071C>G	c.218G>C	r.218g>c	0.0003255
**Pt-7**	RP	*CDHR1*	g.85962879G>A	c.783G>A	r.(642_783del)	0.003052	g.85962879G>A	c.783G>A	r.642_783del	0.003052
**Pt-8**	AS	*CEP78*	g.80863753G>T	c.1033G>T	r.1033g>u	0	g.80863753G>T	c.1033G>T	r.1033g>u	0
**Pt-9**	RP	*EYS*	g.[64430522A>T, 65301854G>T]	c.[3906C>A;9468T>A]	r.[3906c>a,9468u>a]	0.00002564, 0.00002574	g.65229204T>C	c.5644+70912A>G	r.5644_5645ins[5644+70913_5644+71016]	0
**Pt-10**	RP	*EYS*	g.66204881_66204901delinsAAAAG	c.403_423delinsCTTTT	r.403_423delinscuuuu	0	g.66204765G>T	c.539C>A	r.539c>a	0
**Pt-11**	CRD	*EYS*	g.64430522A>T	c.9468T>A	r.9468u>a	0.00002564	g.64430522A>T	c.9468T>A	r.9468u>a	0.00002564
**Pt-12**	RCD	*EYS*	g.66044979A>T	c.1660T>A	r.1660u>a	0.0003055	g.65300716C>A	c.5044G>T	r.5044g>u	0.003442
**Pt-13**	RP	*FAM161A*	g.62063240_62063243del	c.1753_1756del	r.1753_1756del	0	g.62063240_62063243del	c.1753_1756del	r.1753_1756del	0
**Pt-14**	Oguchi disease	*GRM6*	g.178413523G>A	c.1732C>T	r.1732c>u	0.001955	g.178414571dup	c.1355-587dup	r.1354_1355ins[1355-664_1355-590],1154_1250del	0
**Pt-15**	RP	*HGSNAT*	g.43054647G>A	c.1843G>A	r.1843g>a	0.004030	g.43054647G>A	c.1843G>A	r.1843g>a	0.004030
**Pt-16**	RP	*HGSNAT*	g.43037305C>T	c.1030C>T	r.1030c>u	0.00001203	g.43054647G>A	c.1843G>A	r.1843g>a	0.004030
**Pt-17**	RP	*HGSNAT*	g.43054647G>A	c.1843G>A	r.1843g>a	0.004030	c.493+5G>A	c.493+5G>A	r.372_493del	0.00002049
**Pt-18**	RP	*HGSNAT*	g.43054647G>A	c.1843G>A	r.1843g>a	0.004030	g.43054647G>A	c.1843G>A	r.1843g>a	0.004030
**Pt-19**	RP	*HGSNAT*	g.43052991C>T	c.1622C>T	r.1622c>u	0.0001319	g.43054647G>A	c.1843G>A	c.1843g>a	0.004030
**Pt-20**	RP	*PCARE*	g.29293524G>A	c.3604C>T	r.3604c>u	0.000008022	g.29294028_29294029insCCTG	c.3099_3100insCAGG	r.3099_3100inscagg	0.00001370
**Pt-21**	CRD	*RGS9BP*	g.33167752T>G	c.583T>G	r.583u>g	0.0001314	g.33167752T>G	c.583T>G	r.583u>g	0.0001314
**Pt-22**	LCA	*RPGRIP1*	g.21799664_21803187del	c.3238+1118_3339+323del	r.3239_3339del	0	g.21799664_21803187del	c.3238+1118_3339+323del	r.3239_3339del	0
**Pt-23**	RP	*PRPF31*	g.54106454-54133135del	_	_	0	_	_	_	n.a.
**Pt-24**	RP	*USH2A*	g.216270422T>C	c.4758+3A>G	r.4758_4759ins[4758+1_4758+107]	0.001500	g.216523906C>A	c.784+14389G>T	r.784_785ins[784+14397_784+14493]	0.0004781

ABCA4: NG_009073.1; *ATXN7*: NG_008227.1; *C21ORF2*: NG_032952.1; *CDHR1*: NG_028034.1; *CEP78*: NG_053171.1; *EYS*: NG_023443.2; *FAM161A*: NG_028125.1; *GRM6*: NG_008105.1; *HGSNAT*: NG_009552.1; *PCARE*: NG_021427.1; *RGS9BP*: NG_016751.1; *RLBP1*: NG_008116.1; *RPGRIP1*: NG_008933.1; *PRPF31*: NG_009759.1; *USH2A*: NG_009497.2. AS, Alström syndrome; CD, cone dystrophy; CRD, cone rod dystrophy; LCA, Leber congenital amaurosis; MD, macular dystrophy; RCD, rod cone dystrophy; RP, retinitis pigmentosa; TCS, target capture sequencing; STGD1, Stargardt disease; WES, whole exome sequencing. *****With neurodegeneration. [#]

gnomAD allele frequency (AF, accessed on April 27, 2021) is the total AF across all populations. An AF of zero indicates the variant is not identified among the tested alleles in gnomAD.

Supplementary Table 3: Wild-type and mutant *in silico* prediction scores embedded in Alamut for candidate variants

Gene	DNA-variant	c. position splice site	SSFL			Max EntScan			NN SPLICE			Gene Splicer			HSF		
			**WT**	**Mut**	**Delta %**	**WT**	**Mut**	**Delta %**	**WT**	**Mut**	**Delta %**	**WT**	**Mut**	**Delta %**	**WT**	**Mut**	**Delta %**
ABCA4	c.6148-89G>A	c.6148-90	67.6	79.1	**-11.5**	-	-	**-**	-	-	**-**	-	-	**-**	-	-	**-**
ABCA4	c.5460+1315_5460+1317delinsTA	c.5460+1315	70.7	72.5	**-1.8**	-	2.7	**-22.5**	-	0.1	**-10**	-	-	**-**	-	78.2	**-78.2**
CYP4V2	c.214+879_214+882delinsG	c.214+881	-	74.7	**-74.7**	-	5.4	**-45**	-	0.3	**-30**	-	-	**-**	-	77.1	**-77.1**
EYS	c.5644+70912A>G	c.5644+70914	85.5	96.2	**-10.7**	7.1	12.3	**-32.5**	0.9	1.0	**-10**	6.4	7.7	**-6.2**	86.1	96.6	**-10.5**
GRM6	c.1355-587dup	c.1355-589	-	78.4	**-78.4**	5.5	7.3	**-15**	-	0.8	**-80**	2.7	3.5	**-3.3**	73.2	82.0	**-8.8**
HGSNAT	c.493+5G>A	c.493+1	63.3	51.1	**12.2**	6.4	-	**53.3**	0.8	0.0	**80**	0.8	-	**3.3**	-	-	**-**
PDE6B	c.469-776C>G	c.469-775	28.8	87.6	**-58.8**	-	8.4	**-70**	-	1.0	**-100**	-	7.7	**-32**	-	95.1	**-95.1**
RLBP1	c.525+425_525+433delinsATA	c.525+436	57.7	66.7	**-9**	0.6	1.4	**-6.6**	0.0	0.1	**-10**	-	-	-	71.3	76.2	**-4.9**
USH2A	c.784+14389G>T	c.784+14397	87.9	92.9	**-5**	8.7	11.0	**-14.3**	0.6	0.9	**-30**	1.6	4.8	**-15.2**	86.5	88.4	**-1.9**

SSFL, SpliceSiteFinder-Like (0-100); MaxEnScan (0-12 for SDSs_0-16 for SASs); NNSPLICE (0-1); GeneSplicer (0-24 for SDSs_0-21 for SASs); HSF, Human Splicing Finder (0-100).

Supplementary Table 4: SpliceAI scores of candidate variants

Gene	DNA allele	DS_AG	DS_AL	DS_DG	DS_DL	DP_AG	DP_AL	DP_DG	DP_DL
ABCA4	c.6148-89G>A	0.00	0.00	0.12	0.23	3	1	3	1
ABCA4	c.5460+1315_5460+1317delinsTA	-	-	-	-	-	-	-	-
CYP4V2	c.214+879_214+882delinsG	-	-	-	-	-	-	-	-
EYS	c.5644+70912A>G	0.6	0.05	0.66	0.00	-1	-2	-104	-2
GRM6	c.1355-587dup	0.05	0.00	0.06	0.00	-49	-66	3	-70
HGSNAT	c.493+5G>A	0.00	0.22	0.60	0.74	-474	-126	-14	-5
PDE6B	c.469-776C>G	0.00	0.00	0.05	0.01	-207	-405	-1	-97
RLBP1	c.525+425_525+433delinsATA	-	-	-	-	-	-	-	-
USH2A	c.784+14389G>T	0.03	0.00	0.12	0.00	-8	-132	-104	-200

The positions are based on the distance from the variant. DS, delta score; AG, acceptor gain; AL, acceptor loss; DG, donor gain; DL, donor loss; DP, delta position. 500 bp up- and downstream of a variant were included as an input sequence.

Supplementary Table 5: The percentage of each fragment in wild-type and mutant midigenes for candidate variants with multiple mRNA molecules in RT-PCR analysis

	Raw intensity	Fragment size (bp)	Correction factor[#]	Corrected intensity	Percentage
***EYS*_c.5644+70912A>G; p.(Asp1882Glyfs*18)**					
***EYS*_WT_ larger fragment**	21989.4	378	1.38	15939.4	23.6%
***EYS*_WT_ smaller fragment**	51721.5	274	1	51721.5	76.4%
***EYS*_Mut_ larger fragment**	22284.4	378	1.38	16153.2	100%
***GRM6*_c.1355-587dup; p.[Asn451_Ala452ins25,Gly385Alafs*42,=]**					
***GRM6*_ WT_ larger fragment**	60862.7	492	1	60862.7	45.6%
***GRM6*_WT_ smaller fragment**	53110.7	359	0.73	72786.9	54.4%
***GRM6*_Mut_ larger fragment**	33374.9	567	1.15	28960.3	21.0%
***GRM6*_Mut_ middle fragment**	59069.05	492	1	59069	42.0%
***GRM6*_Mut_ smaller fragment**	41692.05	395	0.80	51930.3	37.0%
***USH2A*_c.784+14389G>T; p.(Gly262Aspfs*26)**					
***USH2A*_WT fragment**	3501.2	274	1	3501.2	100%
***USH2A*_Mut_ larger fragment**	11286.1	371	1.35	8335.3	77.1%
***USH2A*_Mut_ smaller fragment**	2483.3	274	1	2483.3	22.9%
***USH2A*_c.4758+3A>G; p.(Gln1586_Gly1587ins5*)**					
***USH2A*_WT fragment**	26776.5	225	1	26776.5	100%
***USH2A*_Mut_ larger fragment**	28628.4	332	1.47	19401.7	78.9%
***USH2A*_Mut_ smaller fragment**	5171.9	225	1	5171.9	21.1%

[#] The size of the wild-type fragment in each variant was utilized as the default for the correction factor. Next, we divided each fragment size to the corresponding wild-type fragment. WT, wild-type; Mut, mutant; bp, base pair.

Supplementary Table 6: The allele frequency of the European (non-Finnish) population, the allele count and number of homozygous individuals in the pathogenic variants identified in 24 solved cases

ID	Gene	DNA allele 1	gnomAD-AF (European (non-Finnish))	Total allele count	Total number of homozygotes	DNA allele 2	gnomAD-AF (European (non-Finnish))	Total allele count	Total number of homozygotes
**Pt-1**	*ABCA4*	**c.6079C>T**	0.0003485	56	0	**c.5196+1137G>A**	0.0001297	3	0
**Pt-2**	*ABCA4*	**c.6743T>C**	0	0	0	**c.4539+2028C>T**	0.00006483	2	0
**Pt-3**	*ABCA4*	**c.5603A>T**	0.06647	11928	364	**c.286A>C**	0.000008804	1	0
**Pt-4**	*ATXN7*	**c.88_117CAG[43]**	0	0	0	**_**	n.a.	n.a.	n.a.
**Pt-5**	*C21ORF2*	**c.218G>C**	0.0006506	90	0	**c.76T>C**	0	0	0
**Pt-6**	*C21ORF2*	**c.218G>C**	0.0006506	90	0	**c.218G>C**	0.0006506	90	0
**Pt-7**	*CDHR1*	**c.783G>A**	0.004903	863	4	**c.783G>A**	0.004903	863	4
**Pt-8**	*CEP78*	**c.1033G>T**	0	0	0	**c.1033G>T**	0	0	0
**Pt-9**	*EYS*	**c.[3906C>A;9468T>A]**	0.00003077,<br>0.00006172	4<br>4	0<br>0	**c.5644+70912A>G**	0	0	0
**Pt-10**	*EYS*	**c.403_423delinsCTTTT**	0	0	0	**c.539C>A**	0	0	0
**Pt-11**	*EYS*	**c.9468T>A**	0.00003077	4	0	**c.9468T>A**	0.00003077	4	0
**Pt-12**	*EYS*	**c.1660T>A**	0.00001558	86	1	**c.5044G>T**	0.00006800	631	9
**Pt-13**	*FAM161A*	**c.1753_1756del**	0	0	0	**c.1753_1756del**	0	0	0
**Pt-14**	*GRM6*	**c.1732C>T**	0.001976	532	1	**c.1355-587dup**	0	0	0
**Pt-15**	*HGSNAT*	**c.1843G>A**	0.005417	1119	4	**c.1843G>A**	0.005417	1119	4
**Pt-16**	*HGSNAT*	**c.1030C>T**	0.00001770	3	0	**c.1843G>A**	0.005417	1119	4
**Pt-17**	*HGSNAT*	**c.1843G>A**	0.005417	1119	4	**c.493+5G>A**	0.00003584	5	0
**Pt-18**	*HGSNAT*	**c.1843G>A**	0.005417	1119	4	**c.1843G>A**	0.005417	1119	4
**Pt-19**	*HGSNAT*	**c.1622C>T**	0.0002336	37	0	**c.1843G>A**	0.005417	1119	4
**Pt-20**	*PCARE*	**c.3604C>T**	0.000008844	2	0	**c.3099_3100insCAGG**	0.00001002	3	0
**Pt-21**	*RGS9BP*	**c.583T>G**	0.00008429	31	0	**c.583T>G**	0.00008429	31	0
**Pt-22**	*RPGRIP1*	**c.3238+1118_3339+323del**	0	0	0	**c.3238+1118_3339+323del**	0	0	0
**Pt-23**	*PRPF31*	**_**	0	0	0	**_**	n.a.	n.a.	n.a.
**Pt-24**	*USH2A*	**c.4758+3A>G**	0.0002025	422	5	**c.784+14389G>T**	0.0007782	15	0

AF, allele frequency. Allele frequency of zero indicates the variant is not reported in the database.”.

Supplementary Table 7: Details of primers synthesized for midigene splice assays of candidate variants

Name	Sequence (5' to 3')	Tm (°C)	GC%	Size (bp)
**ABCA4_ c.6148-89G>A_mut**	gggctcccagatctcatgcctccagaatgaaat	80.1	51.5	10,131
**ABCA4_ c.6148-89G>A_Mut**	atttcattctggaggcatgagatctgggagccc	80.1	51.5	
**ABCA4_ c.6148-89G>A_ validation_R**	gagagtttccgcttgttgc	63.0	52.6	n.a.
**ABCA4_ c.6148-89G>A_RT-PCR**	aagatgctcactggggacac	64.6	55.0	518
**ABCA4_ c.6148-89G>A_RT-PCR**	gaatggtgcccatacatcg	64.3	52.6	
***ABCA4*_BAC_Ex37_Ex40_F**	aggtgtctgctgagcttcttc	58.8	50.0	7,441
***ABCA4*_BAC_Ex37_Ex40_R**	tgctctctcccacacaaaag	59.0	50.0	
**Mut_*ABCA4*_c.5460+1315-17del_F**	tgttcttatccccccttttccagagtctgactctg	76.9	50.0	7,441
**Mut_*ABCA4*_c.5460+1315-17del_R**	cagagtcagactctggaaaggggggataagaaca	76.9	50.0	
**Mut_*ABCA4*_c.5460+1315insTA_F**	tgttcttatccccctactttccagagtctgactctg	75.1	47.2	7,441
**Mut_*ABCA4*_c.5460+1315insTA_R**	cagagtcagactctggaaagtagggggataagaaca	75.1	47.2	
***ABCA4*_Intron 38_F**	ctggggaacacaggacaaat	59.8	50.0	392
***ABCA4*_Intron 38_R**	gggtgcttagcaaatgttgg	60.5	50.0	
***CACNA2D4*_Exon19-23_tag_F**	attcagttccttggccacac	59.9	50.0	6,768
***CACNA2D4*_Exon19-23_tag_R**	caacccagagaaaggagctg	59.9	55.0	
**Mut_*CACNA2D4*_c.2009-3C>T_F**	tcatgcaggcctaaaaccagagtccacattcaggg	81.3	51.4	6,768
**Mut_*CACNA2D4*_c.2009-3C>T_R**	ccctgaatgtggactctggttttaggcctgcatga	81.3	51.4	
***CACNA2D4*_Intron20_F**	acagaccccagtccctttct	59.9	55.0	400
***CACNA2D4*_Intron20_R**	ctcctggcaaagtcctgaac	59.8	55.0	
***CACN2D4*_Exon 19_F**	gcgagttcttttcctgacca	60.4	50.0	345
***CACN2D4*_Exon 23_R**	gctgtccagtaggcttccat	59.3	55.0	
***CYP4V2*_Ex1-Ex3_tag_F**	gtagagcaacctcgcagcac	61.1	60.0	4,848
***CYP4V2*_Ex1-Ex3_tag_R**	ccttcctttccagcctttct	59.8	50.0	
**Mut_*CYP4V2*_ c.214+879del_F**	cgttttacaggctgagcatctaactccaaagcttgaag	77.4	44.7	4,848
**Mut_*CYP4V2*_ c.214+879del_R**	cttcaagctttggagttagatgctcagcctgtaaaacg	77.4	44.7	
**Mut_*CYP2V4*_TAAC>G_F**	cgttttacaggctgagcatgtaactccaaagcttgaag	77.9	44.7	4,848
**Mut_*CYP2V4*_TAAC>G_R**	cttcaagctttggagttacatgctcagcctgtaaaacg	77.9	44.7	
***CYP4V2*_Intron 1_F**	cctggtatttacaactccctgtc	58.9	47.8	365
***CYP4V2*_Intron 1_R**	tgatgatatttggttggcaaag	59.8	36.3	
***CYP4V2*_RT-PCR_Exon 1_F**	gccagtctggtcctgagc	59.5	66.6	320
***CYP4V2*_RT-PCR_Exon 3_R**	aggccaagccatggttcta	60.6	52.6	
***EYS*_Intron 26_tag_F**	ctccccagtttggtttgcta	60.1	50.0	982
***EYS*_Intron 26_tag_R**	gtgtttgcttccctttttgc	59.7	45.0	
**Mut_*EYS*_c.5644+70912A>G_F**	tggtgctgggagtttcctgaggaaaaaaaggacagg	81.2	50.0	982
**Mut_*EYS*_c.5644+70912A>G_R**	cctgtccttttttttcctcaggaaactcccagcacca	81.2	50.0	
***EYS*_Intron 26_F**	aagcccactaggatttctttcc	59.9	45.4	381
***EYS*_Intron 26_R**	gggtaggtgtttgagtgagca	60.2	52.4	
***GRM6*_Ex5-Ex8_tag_F**	gcagcatcctaacaggaaaga	59.5	47.6	3,961
***GRM6*_Ex5-Ex8_tag_R**	tctgacacccaagctggatt	60.6	50.0	

**Mut_*GRM6*_c.1355-587dup_F**	gtggcatcagacggtaatttgcgcatgtgtgac	81.3	51.5	3,961
**Mut_*GRM6*_c.1355-587dup_R**	gtcacacatgcgcaaattaccgtctgatgccac	81.3	51.5	
***GRM6*_Intron 6_F**	ttatgcaaccatcaccacca	60.8	45.0	451
***GRM6*_Intron 6_R**	agttccactcccgggtagata	59.8	52.4	
***GRM6*_RT-PCR_Exon 5_F**	agaacaaccgcaggaacatc	59.2	60.0	492
***GRM6*_RT-PCR_Exon 8_R**	agcagggcagagtagctgag	60.9	55.0	
***HGSNAT*_Ex3-Ex5_tag _F**	acaattccatagtgacacagtga	57.1	39.1	3,959
***HGSNAT*_Ex3-Ex5_tag_R**	ccagtcccttctccacattg	60.5	55.0	
**Mut_*HGSNAT*_c.493+5G>A_F**	gaaaatcagcagagaacatatatgtacgaaggttactatcaactg	73.5	35.6	3,959
**Mut_*HGSNAT*_c.493+5G>A_R**	cagttgatagtaaccttcgtacatatatgttctctgctgattttc	73.5	35.6	
***HGSNAT*_Exon 4_F**	tccgtacccaggaacatgtatt	60.5	45.4	517
***HGSNAT*_Exon 4_R**	attacaagcatgagccaccat	59.5	42.8	
***HGSNAT*_RT-PCR_Exon 3_F**	tcaggttctggtaaacgttcct	60.0	45.4	293
***HGSNAT*_RT-PCR_Exon 5_R**	caatgatgacagcaagaccaat	60.0	40.9	
***USH2A*_Intron 4_tag_F**	agcattaagcctgtgggtgt	59.6	50.0	935
***USH2A*_Intron 4_tag_R**	gaggaggtgccacacacttt	60.2	55.0	
**Mut_*USH2A*_c.784+14389G>T_F**	ccatgcaagtctgaaacacaaaagggcagtcatca	80.5	45.7	935
**Mut_*USH2A*_c.784+14389G>T_R**	tgatgactgcccttttgtgtttcagacttgcatgg	80.5	45.7	
***USH2A*_Intron 4_F**	ctcactcctcccaccaatgt	59.9	55.0	318
***USH2A*_Intron 4_R**	ttcccaacagtctcccaaag	60.0	50.0	
***RLBP1*_Exon_6_F**	tgcccaagcttatgatgatg	59.6	45.0	4031
***RLBP1*_Exon_7_R**	ccttcgtttccaggtttgaa	60.1	45.0	
***RLBP1*_intron_6_F**	tgccacaaaatctctatcatcga	59.5	55.0	386
***RLBP1*_intron_6_R**	tttcactcctggggtctgtc	59.2	55.0	
***RLBP1*_RT-PCR_ Exon 6_F**	acaagtatggccgagtggtca	60.0	55.0	226
***RLBP1*_RT-PCR_ Exon 7_R**	agcatgtccaccatcttcct	59.5	50.0	
***PDE6B*_ Intron 1_F**	caggaaatgaggagcaggaa	60.3	50.0	941
***PDE6B*_ Intron 1_R**	cttcctggatgcctgttgag	60.8	55.0	
***PDE6B*_Intron 1_conf_F**	gtccacctgggtcactgc	60.1	66.6	396
***PDE6B*_Intron 1_conf_R**	tttcactcctggggtctgtc	60.1	55.0	
***RHO*_Exon 3_F**	cggaggtcaacaacgagtct	64.7	55.0	-
***RHO*_Exon 5_F**	atctgctgcggcaagaac	64.7	55.6	140
***RHO*_Exon 5_R**	aggtgtaggggatgggagac	64.5	60.0	

The sequence of all designed primers for midigene assays with GC content and melting temperature, tm(°C) and size of PCR products. bp, base pairs; n.a., not applicable

Supplementary Note 1: Details of prior genetic testing approached for cases in this study

Whole exome sequencing (WES) was performed at BGI-Europe (Copenhagen, Denmark). In short, by application of Agilent's SureSelectXT Human all Exon V5 (Agilent Technologies, Santa Clara, CA), the exome was enriched initially followed by next-generation sequencing using an Illumina HiSeq 4000 sequencer (Illumina, Inc. San Diego, CA) to a mean sequence depth of at least 75-fold. The reads were aligned to the human reference genome (hg19) using Burrows-Wheeler Aligner. Subsequently, nucleotide and copy number variant calling were performed with the Genome Analysis Toolkit and CoNIFER 0.2.0, respectively[1]. Ultimately, variants were annotated using a bespoke in-house annotation strategy[2]. For the Israeli cases, the targeted capture sequencing was performed by molecular inversion probes (MIPs) with 5-bp molecular tags incorporating 108 genes associated with inherited retinal diseases. Sequencing was performed by Illumina NextSeq 500 system and the reads were aligned to a human reference sequence (hg19) via the Burrows-Wheeler Aligner (BWA) v.0.6.2. The variant calling tools and annotation pipeline were similar to the WES method[3]. Targeted capture sequencing of Irish cases was performed with the Nimblegen SeqCap EZ kit (Roche Ireland Ltd., Dublin, Ireland), incorporating the exonic regions of 254 genes associated with retinopathies followed by sequencing the enriched region using an Illumina MiSeq (Illumina Inc., San Diego, CA, USA) with the average read coverage of 125× per captured region[4,5]. The reads were aligned to the human reference genome (hg38) using Burrows-Wheeler Aligner 0.7.15. Subsequently, nucleotide, copy number and structural variant calling were performed with using Freebayes 1.1.0 (arXiv:1207.3907), CoNIFER[1] and Manta[6] respectively.

Supplementary References

1 Krumm, N. *et al.* Copy number variation detection and genotyping from exome sequence data. *Genome Res* **22**, 1525-1532, doi:10.1101/gr.138115.112 (2012).

2 Verbakel, S. K. *et al.* The identification of a RNA splice variant in TULP1 in two siblings with early-onset photoreceptor dystrophy. *Mol Genet Genomic Med* **7**, e660, doi:10.1002/mgg3.660 (2019).

3 Weisschuh, N. *et al.* Molecular and clinical analysis of 27 German patients with Leber congenital amaurosis. *PLoS One* **13**, e0205380, doi:10.1371/journal.pone.0205380 (2018).

4 Dockery, A. *et al.* Target 5000: Target Capture Sequencing for Inherited Retinal Degenerations. *Genes (Basel)* **8**, doi:10.3390/genes8110304 (2017).

5 Whelan, L. *et al.* Findings from a Genotyping Study of Over 1000 People with Inherited Retinal Disorders in Ireland. *Genes (Basel)* **11**, doi:10.3390/genes11010105 (2020).

6 Chen, X. *et al.* Manta: rapid detection of structural variants and indels for germline and cancer sequencing applications. *Bioinformatics* **32**, 1220-1222, doi:10.1093/bioinformatics/btv710 (2016).

Supplementary Note 2: Details of the automated prioritization pipeline

```
# Load the required packages
library(stringr)
library(data.table)
# Define the IRD WGS filtering function
IRD_WGS_filtering <- function(sample, path){
# Perform filtering for sv file
svfile <- fread(file = paste0(path,
sample, "/00_raw.unfiltered/", "/", sample, ".sv.segments.txt"),
sep = '\t', fill = TRUE)
svfile_filtered <- svfile[
(str_detect(svfile$DISEASE, "BLIND") &
(svfile$SVTYPE != "BND") &
str_detect(svfile$filter, "PASS")
)
]
write.csv(svfile_filtered,
file = paste0(path,
sample, "/01_filtered/", sample,
".sv.segments_filtered.txt"))
# Perform filtering for cnv file
cnvfile <- fread(file = paste0(path,
sample, "/00_raw.unfiltered/", "/", sample, ".cnv.segments.txt"),
sep = '\t', fill = TRUE)
cnvfile_filtered <- cnvfile[
(str_detect(cnvfile$DISEASE, "BLIND")
)
]
write.csv(cnvfile_filtered,
file = paste0(path,
sample, "/01_filtered/", sample,
".cnv.segments_filtered.txt"))
# Perform filtering for snv.IRDextract file
snvIRD <- fread(file = paste0(path,
sample, "/00_raw.unfiltered/", "/", sample,
".WGS.IRDextract.hcdiffs.txt"),
sep = '\t', fill = TRUE)
snvIRD_filtered <- snvIRD[
((snvIRD$`NonCausative - Frequency` <= 5.0) &
(snvIRD$`gnomAD-E AF` <= 5.0) &
(snvIRD$`gnomAD-G AF` <= 5.0) &
(str_detect(snvIRD$`Protein Effect`,
paste0("stop\\_gained|frameshift\\_variant|stop\\_lost|",
"inframe\\_deletion|inframe\\_insertion|stop\\_gained|",
"splice\\_region\\_variant")) |
(str_detect(snvIRD$`Protein Effect`,"missense\\_variant") &
(snvIRD$phyloP >= 2.7)) |
(str_detect(snvIRD$`Protein Effect`,"missense\\_variant") &
(snvIRD$CADD_PHRED >= 15)) |
(str_detect(snvIRD$`Protein Effect`,"missense\\_variant") &
(snvIRD$`Grantham Score` >= 80)) |
(str_detect(snvIRD$`Protein Effect`,"synonymous\\_variant") &
(snvIRD$phyloP >= 2.7) &
(snvIRD$CADD_PHRED >= 15)) |
str_detect(snvIRD$`Gene component`,
paste0("INTRON\\_REGION|SA\\_SITE\\_CANONICAL|SD\\_SITE\\_CANONICAL|",
"CODING\\_SPLICE\\_SITE_REGION|NONCODING\\_SPLICE\\_SITE\\_REGION"
))
)
)
]
write.csv(snvIRD_filtered,
file = paste0(path,
sample, "/01_filtered/", sample,
".WGS.IRDextract.hcdiffs_filtered.txt"))
# Perform filtering for snv file
snv <- fread(file = paste0(path,
sample, "/00_raw.unfiltered/", "/", sample, ".snv.hcdiffs.txt"),
sep = '\t', fill = TRUE)
```

```
snv_filtered <- snv[
((snv$`NonCausative - Frequency` <= 5.0) &
(snv$`gnomAD-E AF` <= 5.0) &
(snv$`gnomAD-G AF` <= 5.0) &
(str_detect(snv$`Protein Effect`,
paste0("stop\\_gained|frameshift\\_variant|stop\\_lost|",
"inframe\\_deletion|inframe\\_insertion|stop\\_gained|",
"splice\\_region\\_variant")) |
(str_detect(snv$`Protein Effect`,"missense\\_variant") &
(snv$phyloP >= 2.7)) |
(str_detect(snv$`Protein Effect`,"missense\\_variant") &
(snv$CADD_PHRED >= 15)) |
(str_detect(snv$`Protein Effect`,"missense\\_variant") &
(snv$`Grantham Score` >= 80)) |
(str_detect(snv$`Protein Effect`,"synonymous\\_variant") &
(snv$phyloP >= 2.7) &
(snv$CADD_PHRED >= 15)) |
str_detect(snv$`Gene component`,
paste0("SA\\_SITE\\_CANONICAL|SD\\_SITE\\_CANONICAL|",
"CODING\\_SPLICE\\_SITE_REGION|NONCODING\\_SPLICE\\_SITE\\_REGION"
))
)
)
]
write.csv(snv_filtered,
file = paste0(path,
sample, "/01_filtered/", sample,
".snv.hcdiffs_filtered.txt"))

}
```
